# Size-tuneable and immunocompatible polymer nanocarriers for drug delivery in pancreatic cancer†

**DOI:** 10.1039/d2nr00864e

**Published:** 2022-04-19

**Authors:** Andrea Bistrović Popov, Francesca Melle, Emily Linnane, Cristina González-López, Ishtiaq Ahmed, Badri Parshad, Christoph O. Franck, Hassan Rahmoune, Frances M. Richards, Daniel Muñoz-Espín, Duncan I. Jodrell, David Fairen-Jimenez, Ljiljana Fruk

**Affiliations:** BioNano Engineering Lab, Department of Chemical Engineering and Biotechnology, University of Cambridge Philippa Fawcett Drive Cambridge CB3 0AS UK lf389@cam.ac.uk; The Adsorption & Advanced Materials Laboratory (A2ML), Department of Chemical Engineering and Biotechnology, University of Cambridge Philippa Fawcett Drive Cambridge CB3 0AS UK; CRUK Cambridge Centre Early Detection Program, Department of Oncology, Hutchison/MRC Research Centre, University of Cambridge Cambridge CB2 0RE UK; Department of Chemical Engineering and Biotechnology, University of Cambridge Philippa Fawcett Drive Cambridge CB3 0AS UK; Cancer Research UK Cambridge Institute, University of Cambridge, Li Ka Shing Centre Cambridge CB2 0RE UK; Translational Medicine, Oncology R&D, Astra Zeneca Cambridge CB4 0WG UK; Department of Oncology, University of Cambridge, Hutchison/MRC Research Centre Cambridge Biomedical Campus Cambridge CB2 0XZ UK

## Abstract

Nanocarriers have emerged as one of the most promising approaches for drug delivery. Although several nanomaterials have been approved for clinical use, the translation from lab to clinic remains challenging. However, by implementing rational design strategies and using relevant models for their validation, these challenges are being addressed. This work describes the design of novel immunocompatible polymer nanocarriers made of melanin-mimetic polydopamine and Pluronic F127 units. The nanocarrier preparation was conducted under mild conditions, using a highly reproducible method that was tuned to provide a range of particle sizes (<100 nm) without changing the composition of the carrier. A set of *in vitro* studies were conducted to provide a comprehensive assessment of the effect of carrier size (40, 60 and 100 nm) on immunocompatibility, viability and uptake into different pancreatic cancer cells varying in morphological and phenotypic characteristics. Pancreatic cancer is characterised by poor treatment efficacy and no improvement in patient survival in the last 40 years due to the complex biology of the solid tumour. High intra- and inter-tumoral heterogeneity and a dense tumour microenvironment limit diffusion and therapeutic response. The Pluronic-polydopamine nanocarriers were employed for the delivery of irinotecan active metabolite SN38, which is used in the treatment of pancreatic cancer. Increased antiproliferative effect was observed in all tested cell lines after administration of the drug encapsulated within the carrier, indicating the system's potential as a therapeutic agent for this hard-to-treat cancer.

## Abstract 2

Pluronic-polydopamine nanocarriers were used to evaluate the effect of particle size on immunocompatiblity and cell uptake in pancreatic cancer. The drug-loaded carriers exhibited enhanced antiproliferative effects in this hard-to-treat cancer.

## Graphical abstract

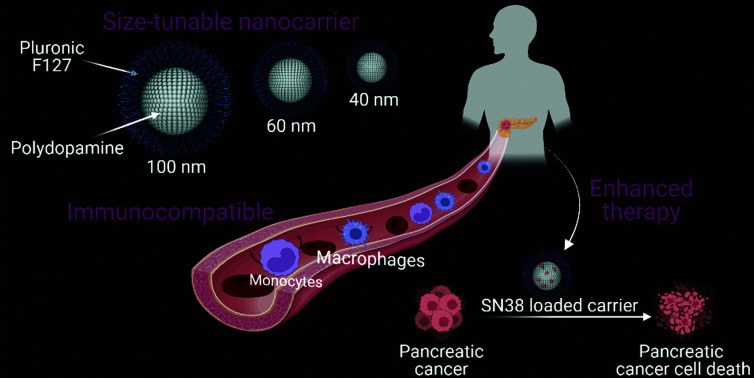

## Introduction

Since the approval of liposomal doxorubicin for the treatment of Kaposi's sarcoma in 1995,^1^ nanomaterials have been extensively explored in the design of drug nanocarriers. Over 50 nanoformulations, including liposomes, polymers and albumin nanoparticles (NPs), have already been approved for clinical use, mostly in cancer therapy.^2,3^ However, the discrepancy between the number of pre-clinical studies on nanoformulations compared to those which make clinical translation was recently addressed in a number of essays and opinion articles.^4–8^ The authors outlined several specific challenges, such as insufficient clarity in regulatory guidelines, the need for more relevant validation strategies to cover the disease heterogeneity and the ability to fine-tune the carrier systems based on the tumour biology. Despite these challenges, nanocarriers show distinct therapeutic advantages by improving the pharmacokinetic profile of poorly soluble drugs as well as various pharmacological parameters, including clearance rate and peak drug concentration.^8^ Additionally, the recent development of lipid NP-stabilized mRNA vaccines for Covid-19,^9^ as well as better understanding of the biological and molecular aspects of diseases achieved through multidisciplinary collaborative efforts are changing the nanomedicine field.

Physicochemical properties such as size, charge and hydrophobicity, as well as mechanical characteristics, such as softness and rigidity, of nanocarriers are crucial for their transport and tissue penetration. Previous studies have shown that smaller particles (≤100 nm) readily diffuse within solid tumours, but larger particles (100–150 nm) circulate longer in blood, leading to increased accumulation in the target tissue.^10–12^ For most polymer nanocarriers, size control is achieved by changing the chemical composition of the core structure or the number and length of the surface groups, which often results in inconsistent reports on the nanocarrier size impact.^13^ The most commonly employed surface coating is polyethylene glycol (PEG) known to stabilise NPs and minimise their interactions with the immune system and blood components.^14^ However, the functionalisation of nanomaterials with PEG moieties can hinder cell internalization and has shown an immune-modulating effect indicated by the presence of anti-PEG antibodies.^15–17^ Therefore alternative surface coatings are being explored.

In this study, biopolymer NPs containing Pluronic as an alternative to PEG were designed. Pluronic is an amphiphilic triblock copolymer composed of two hydrophilic polyethylene oxide (PEO) blocks and a hydrophobic polypropylene oxide (PPO) segment. Versatility of the chemical composition and simple functionalisation make Pluronic polymers a promising alternative to PEG for biomedical applications. For example, it has been shown that the interactions with cells can be altered by adjusting the composition (PEO/PPO ratio) of Pluronic, which impacts its physical and chemical properties.^18,19^ As in the case of PEG, the addition of Pluronic to NPs leads to a net-neutrally charged hydrophilic surface, which can facilitate penetration through the tumour extracellular matrix (ECM).^20–22^ The interest in the amphiphilic polymer was further enhanced with phase III clinical trials of doxorubicin-encapsulated Pluronic L61 and F127 micelles (SP1049C) for treatment of gastric and oesophagus cancer.^23^ In addition, Pluronic polymers were shown to be effective sensitisers in multidrug resistant (MDR) cancer cells through different mechanisms, such as inhibition of the drug efflux transporter *P*-glycoprotein or by micro-viscosity modification of the cellular membrane.^24^ All of these properties make them well-suited for application in drug delivery to pancreatic ductal adenocarcinoma (PDAC), a highly heterogenous type of cancer characterised by high level of multidrug resistance.

Even though the treatment of PDAC mainly relies on chemotherapy, as only 10–20% of patients can undergo surgery, it shows resistance to both chemotherapy and radiotherapy resulting in a 5-year survival rate below 8%.^25,26^ PDAC is the fifth most common cause of cancer death in the UK without significant improvement in outcome over the last 40 years.^27,28^ This dire prognosis is attributed to the lack of specific symptoms, early onset of metastasis, complex tumour biology and a dense, heterogenous microenvironment, known as the stroma. The stromal tissue accounts for up to 80% of the total tumour volume and contributes to the high density, stiffness and interstitial pressure, acting as a shielding physical barrier to therapeutic delivery.^29,30^

**Fig. 1:**
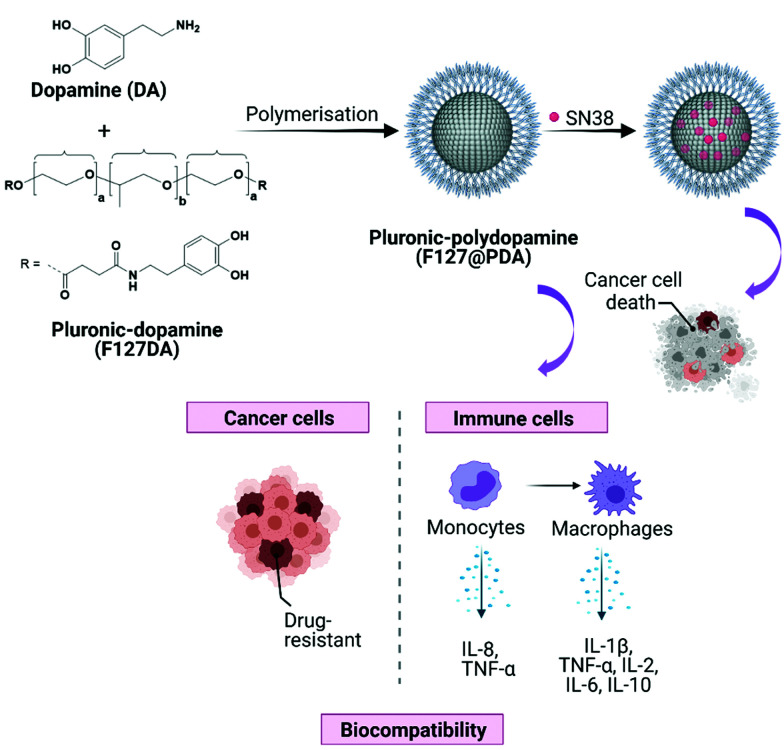
Schematic illustration displaying the synthesis and evaluation of biocompatible F127@PDA NPs for drug delivery application.

Herein, the reproducible synthesis of size-tuneable Pluronic F127-polydopamine (F127@PDA) nanocarriers and the evaluation of sub-100 nm carriers on immunocompatibility and cell uptake in different PDAC cells is reported (Fig. 1). The reported nanocarriers incorporate Pluronic within the core structure trough co-polymerisation, which results in highly stable and reproducible nanoparticles. To improve the rational design and validation of the carrier prior to animal studies, *in vitro* studies were conducted using four pancreatic cell lines (AsPC-1, BxPC-3, MIA PaCa-2 and PANC-1), which differ in *KRAS* and *TP53* mutation status, variations in ECM production as well as factors driving endocytic pathways of uptake, addressing the heterogeneity of PDAC. Additionally, we loaded the carriers with irinotecan active metabolite SN38, used in PDAC treatment, and found enhanced antiproliferative effect in all PDAC cells upon administration of the SN38 loaded carrier. To our knowledge, this is the first report demonstrating the design of Pluronic F127 polydopamine nanocarriers and their use for drug delivery of this hard to treat cancer.

## Experimental

All reagents unless otherwise stated were purchased from Sigma Aldrich (UK), Acros Organics (UK) or TCI chemicals (UK) and used without further purification. ^1^H NMR spectra were recorded on a 400 MHz DCH Cryoprobe Spectrometer in CDCl3 and DMSO-d_6_. UV-Vis absorption spectra were obtained on an Agilent Cary 300 Spectrophotometer. Fluorescence emission spectra were obtained using a Varian Cary Eclipse Fluorescence Spectrophotometer using excitation and emission splits of 5 nm. DLS and zeta potential measurements were recorded using a Zetasizer Nano ZS instrument (Malvern Panalytical, UK) with a sample concentration of 0.05 mg mL^−1^. All Measurements were conducted three times with 15 subruns for each sample. Error bars represent the standard deviation of three measurements. Zeta potential was measured in a folded capillary Zeta cell DTS1070 (Malvern, UK). FTIR spectroscopy was carried out using a Bruker Tensor 27 spectrometer with samples pressed into KBr pellets. Lyophilisation was carried out using a Telstar LyoQuest benchtop freeze dryer (0.008 mBar, −70 °C).

### Synthesis and characterisation of F127@PDA NPs

#### General procedure for the synthesis of Pluronic-polydopamine (F127@PDA)

Trizma-base (22.5 mg) was dissolved in 2.5 mL Milli-Q water and added to a mixture of ethanol and Milli-Q water (30 mL) and stirred for 30 min at room temperature. Dopamine hydrochloride (dissolved in 1 mL Milli-Q water) and F127DA (dissolved in 1 mL ethanol) were mixed and sonicated before being added dropwise to the reaction mixture. The mixture was left to stir overnight at room temperature resulting in a dark brown solution. The reaction mixture was washed with Milli-Q water using Vivaspin 20 (Satorius, UK) centrifugal concentrators (30 kDa MWCO) for 15 min at 4000 rpm until the supernatant was colourless. The obtained particles were diluted in Milli-Q water, frozen in liquid nitrogen and lyophilised to yield a dark brown powder.

For comparison, samples with varying molar ratio of DA : F127DA or varying volume percent of ethanol in the reaction mixture were prepared, while keeping the other synthesis parameters the same (Table S1, ESI†).

To investigate the formation kinetics of F127@PDA NPs with varying ethanol percentage, the absorbance changes at 400 nm were measured with UV-Vis and hydrodynamic diameter using Zetasizer Nano. At each time point 50 µL of the reaction solution was taken and the absorbance was measured.

#### Colloidal stability of F127@PDA NPs

The colloidal stability of NPs, with and was evaluated in deionized water, PBS (1×, pH = 5.5–8.5), phenol-red free DMEM and DMEM + 10% FBS. PBS was adjusted to different pH by adding HCl or NaOH. Solutions containing 900 μL of solvent and 100 μL of 1.0 mg mL^−1^ sample were incubated for 72 hours at 37 °C. After 72 hours hydrodynamic diameter, zeta potential and UV-Vis were measured.

#### General procedure for fluorescent labelling of F127@PDA

F127@PDA NPs (5 mg) was dissolved in 10 mM Tris buffer (5 mL) and Fluorescein-TEG-NH_2_ (10 mg, 2 wt eq.) dissolved in 0.5 mL ethanol was added dropwise. The mixture was protected from direct sunlight and stirred over night at room temperature. Excess Fluorescein-TEG-NH_2_ was removed washing with Milli-Q water using Vivaspin 20 (Satorius, UK) centrifugal concentrators (10 kDa MWCO) for 15 min at 4000 rpm until the supernatant was colourless, followed by 3 days dialysis against water with 12–14 kDa MWCO dialysis bags.

### *In vitro* evaluation of F127@PDA NPs

#### Cell lines and growth conditions

Human pancreatic cancer cell lines AsPC-1, BxPC-3, MIA PaCA-2 and PANC-1 were purchased from American Type Culture Collection (ATCC). MIA PaCa-2 and PANC-1 were grown in DMEM (Sigma, UK) supplemented with 10% FBS. AsPC-1 and BxPC-3 cells were cultured using RPMI-1640 supplemented with 10% FBS. All cell lines were cultured in a humidified environment at 37 °C with 5% CO_2_. All cell lines were routinely tested to confirm the absence of Mycoplasma and verified by STR profile. *In vitro* experiments were conducted with 60% to 80% confluent cultures at passage number between 5 and 15.

#### Live cell cytotoxicity studies

All NPs were tested for cytotoxicity studies in AsPC-1, BxPC-3, MIA-PaCa-2 and PANC-1. Cells were seeded into 96-well plates at a concentration of 2000 cells per well, in 100 µl of complete growth medium and incubated at 37 °C, 5% CO_2_ for 24 h. After overnight incubation, the cells were treated with different concentrations of F127@PDA-40, F127@PDA-60 and F127@PDA-100 (0.01–100 µg mL^−1^) dissolved in complete cell media containing 0.1% water. The plates were then inserted into the IncuCyte®S3 Live Cell Analysis System (Sartorius) for real-time imaging. Treated plates were imaged every 3 h for 72 h under cell culture conditions with 10× objective using the brightfield channel. Mean cell confluence was calculated using the images taken from 3 random fields of view per well using the IncuCyte S3 v2017A software. All Incucyte experiments were performed in triplicate in three independent experiments. Relative confluence values were obtained by normalizing each value to the time zero value in each sample and normalized to the untreated control sample.1
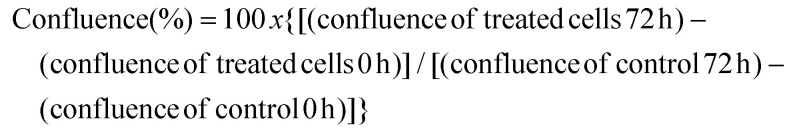


#### MTS cytotoxicity studies

Additionally, the effect of F127@PDA NPs on the viability of AsPC-1, BxPC-3, MIA PaCa-2 and PANC-1 using MTS assay (Promega, USA.) Cells were seeded into clear 96-well plates containing 2000 cells per well in 100 µL complete growth medium and cultured for 24 h at 37 °C and 5% CO_2_. Subsequently, cells were treated with varying concentrations of F127@PDA_40, F127@PDA_60 and F127@PDA_100 (0.01–100 µg mL^−1^) dissolved in complete growth media containing 0.1% water. After further 72 h incubation at 37 °C and 5% CO_2_, 20 µL of CelTiter 96® AQ_ueous_ One Solution (Promega, USA) was added into each well and incubated at 37 °C, 5% CO_2_ for 1–4 h, according to the manufacturer's instruction. The absorbance of each well was measured at 490 nm using a Spark plate reader (TECAN, CH). Control measurements included negative control of cells with DMEM, cells with DMEM containing 0.1% water, cell-free culture media (blank) and cell-free sample dilutions in culture media to evaluate potential sample interferences with MTS reagent. All experiments were conducted in biological triplicates. The percentage cell viability was calculated according to the following:2
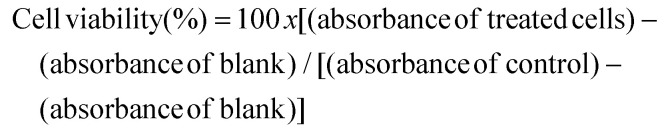


### Immunocompatibility evaluation of F127@PDA NPs

#### Viability test on acute monocyte leukaemia cell line (THP-1)

THP-1 cells were kindly provided by Dr Hassan Rahmoune (Department of Chemical Engineering and Biotechnology, University of Cambridge, UK) and maintained in RPMI-1640 medium with l-glutamine and sodium bicarbonate (Sigma), supplemented with 10% FBS (Gibco) and 1% penicillin–streptomycin (Thermo Fisher Scientific). THP-1 differentiation was induced by phorbol-12-myristate 13-acetate, 100 nM (PMA, Sigma-Aldrich) for 48 h. After differentiation the medium was replenished with full growth media and the cells were incubated for additional 24 hours at 37 °C and 5% CO_2_. To determine the effect of F127@PDA NPs on the viability of THP-1 cells and THP-1 differentiated cells were seeded into a 96-well plate (3000 cells per well) and incubated with varying concentrations of F127@PDA_40, F127@PDA_60 and F127@PDA_100 (0.01–100 µg mL^−1^) for 72 hours. Following incubation, MTS assay was performed as described in the previous section.

#### Cytokine analysis in THP-1 and THP-1 M(0)

The cytokine analysis was conducted according to the procedure described by Zhu *et al.*^31^ Briefly, THP-1 and THP-1 M(0) cells (1 × 10^5^ cells per mL) were seeded in a 24-well plate and treated with 10 μg mL^−1^F127@PDA_40, F127@PDA_60 and F127@PDA_100 for 24 h. Lipopolysaccharide (LPS, 10 ng mL^−1^) was used as a positive control. After incubation 1 mL cell media form individual cells was centrifuged at 1000 rpm for 5 min and the supernatant was collected and kept at −80 °C for cytokine analysis. The quantification of multiple cytokines in the samples was conducted *via* Meso Scale Discovery (MSD) multiplex assay platform. The MSD assay is an ultrasensitive electrochemical luminescence immunoassay performed on the MesoScale Diagnostics Sector Imager 6000. The samples were analysed at the Core Biochemical Assay Laboratory (NHS Cambridge University Hospitals; UK).

### Immunofluorescence staining of PDAC cell lines

Cells were seeded onto 96-well treated plates (PerkinElmer) at 20% confluence. Cells were left for 24–72 h before incubation with CellMask™ deep red stain (ThermoFisher) and then imaging live or fixing with 4% paraformaldehyde (PFA) and washed three times in PBS. For fixed cell samples, cells were blocked in 2% w/v bovine serum albumin (BSA) in PBS for 30 min before incubation with primary antibodies for 1 h at room temperature. Cells were imaged using the Operetta spinning disk confocal microscope (PerkinElmer) using the 63× water objective. Primary antibodies used for immunofluorescence were mouse anti-LAMP-1 (BD Bioscience), mouse anti-EEA-1 (BD Bioscience), Cis-Golgi (Abcam), mouse anti-alpha tubulin (DM1A, Cell Signalling) and rat anti-tubulin (Alexa Fluor® 647, Abcam). Secondary antibodies used were donkey anti-rabbit (Alexa Fluor® 488) and goat anti-mouse (Alexa Fluor® 555) sourced from Abcam. Nuclei were stained with Hoechst 33342 (Thermo Fisher).

### Confocal imaging

Cells were seeded into a glass bottom dish (MatTek Life Science, US) at concentration of 200 000 cells per ml and incubated at 37 °C for 24 h, then treated with different F127@PDA@Fl NPs at different concentrations for 24 h at 37 °C. After 3 washes with 1× PBS the cells were stained with CellMask™ Deep Red (Thermo Fischer) Plasma membrane stain and Hoechst 33342 (Thermo Fisher) according to the manufacturer's instructions. Cells were then washed gently with PBS (1×) three times and imaged using confocal microscope (Axio Observer Z1 LSM 800, Zeiss). Zen software (Zeiss) was used for the acquisition image processing.

### Flow cytometry analysis

Cells were seeded in 6-well plates at a density of 2 × 10^5^ cells per well and cultured for 24 h. The next day cells were treated with various concentrations of F127@PDA@Fl_40, F127@PDA@Fl_60 and F127@PDA@Fl_100 NP solutions prepared in the culture media (10 μg mL^−1^, 20 μg mL^−1^ or 50 μg mL^−1^) and incubated for 24 h. After the treatment, cells were washed three times with 1× PBS to remove residual NPs both in culture media and on the cell surfaces, they were detached with 0.25 mL TrypLE (Thermo Fischer, UK) and centrifuged for 5 min at 300*g*. 1 mL of FACS buffer (PBS with 4% FBS) was added to the cells and 10 μL of 10 μg mL^−1^ DAPI stock solution. The cells were kept at 4 °C until flow cytometry analysis. Flow cytometry was carried out on a Canto II flow cytometer (BD Biosciences) using 355 and 488 lasers. 10 000 events were acquired for each sample. FlowJo software (version 10.2) was used for data analysis. Briefly, the live single-cell population was gated in a plot of FSC *vs.* SSC after excluding cell debris and doublets a histogram from the FITC channel for the single-cell population was obtained and analysed.

### Drug loading and release studies

To assess the loading capacity, absorption of SN38 was investigated adapting a method by Wang *et al.*^32^ A suspension of 10 mg F127@PDA NPs (1 mg mL^−1^) and 2.5 mg SN38 (0.25 wt eq.) in DMSO : H_2_O = 1 : 10 (10 mL) were sonicated for 30 min, followed by stirring at room temperature for 72 hours. To remove the free drug, the reaction mixture was first centrifuged at 2000 rpm for 5 min. The supernatant was collected and washed with Milli-Q water using Vivaspin 20 (Satorius, UK) centrifugal concentrators (10 kDa MWCO) for 15 min at 4000 rpm. The obtained particles were diluted in Milli-Q water, frozen in liquid nitrogen and lyophilized to yield a dark brown powder. The loading content of SN38 within the NPs was determined using UV-Vis. The absorbance of SN38@F127@PDA NPs at 382 nm was deducted by the F127@PDA absorbance and the loading content was calculated according to the following equation: (3).3
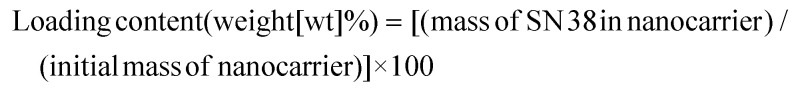


Next, drug release was measured in PBS (1×, pH 7.4). 2 mL of 1 mg mL^−1^SN38@F127@PDA NPs were dissolved in PBS (1×, pH 7.4) and placed in a dialysis bag (MWCO 12–14 kDa) and dialyzed against PBS (25 mL) in an incubator shaker at 37C. 1 mL of the media was removed and replaced with fresh media at different time intervals. The amount of the released drug was quantified using HPLC as described by Xuan *et al.*^33^ Briefly, the HPLC analysis was conducted on an Agilent 1260 Infinity Quaternary LC equipped using an Agilent Zorbax SB-C18 (4.6 mm × 250 mm, 5 μm) analytical column. The mobile phase consisted of a mixture of NaH_2_PO_4_ (pH 3.1, 25 mM) and acetonitrile (50 : 50, v/v) with a 1 mL min^−1^ flow rate. SN38 concentration was detected at 265 nm and an external calibration curve for both SN38 forms (carboxylic acid and lactone) were used for quantification (Fig. S16, ESI†).

### Drug release *in vitro*

Cells were seeded into 96-well plates at concentration of 2000 cells per well, in 100 µl of complete growth medium and incubated at 37 °C, 5% CO_2_ for 24 h. After overnight incubation, the cells were treated with SN38 and the same concentration of SN38@F127@PDA using various concentrations (0.0001 μM – 1 μM). The plates were inserted into the IncuCyte®S3 Live Cell Analysis System (Sartorius) for real-time imaging. Treated plates were imaged every 3 h for 72 h under cell culture conditions with 10× objective using the brightfield channel. Average cell confluence was calculated as described in the previous section. In addition to live cell imaging after 72 h incubation in the IncuCyte®S3 MTS assay was performed as described in the section above. Molar concentration of SN38 in SN38@F127@PDA NPs in a 1 mg mL^−1^ solution was calculated according to the following:4
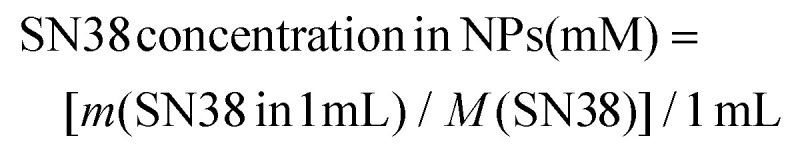


### Statistical analysis

Experiments were independently repeated at least in triplicates unless otherwise noted and all data presented as mean ± standard deviation. All statistical analysis was done with GraphPad Prism 9 software (GraphPad Software, San Diego, CA, USA). Significance levels are defined as the following: ns for *p* > 0.05, * for *p* ≤ 0.05, ** for *p* ≤ 0.01, *** for *p* < 0.001, and **** for *p* < 0.0001.

## Results and discussion

### Synthesis and characterization of F127@PDA

**Fig. 2:**
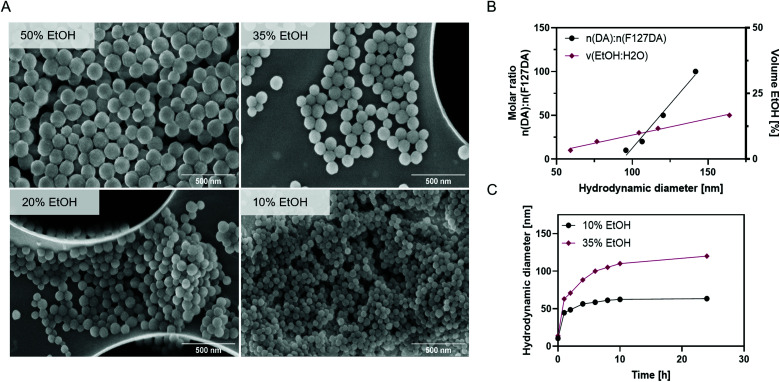
Characterization of F127@PDA. SEM images of F127@PDA NPs prepared in a 20 : 1 molar ratio of DA : F127DA with 10, 20, 35 and 50% ethanol in water (A). Linear correlation between the molar ratio of DA : F127DA and the solvent ratio of EtOH : H_2_O with the hydrodynamic diameter of F127@PDA NPs (B). Hydrodynamic diameter evolution over time during the formation of F127@PDA_40 and F127@PDA_100 prepared in a 20 : 1 molar ratio of DA : F127DA with 10% and 35% EtOH in the reaction mixture, respectively (C).

Melanin-mimetic F127@PDA NPs were prepared by oxidation and self-co-polymerization of dopamine hydrochloride (DA) and Pluronic F127-dopamine (F127DA) monomer (Fig. 1). Melanin-like polymers are highly biocompatible, act as radical scavengers and neuroprotection agents,^34,35^ as well as possessing excellent photothermal^36–38^ and photoacoustic^39–41^ properties, all of which makes them a particularly promising candidate for drug delivery and diagnostics. The F127DA monomer was first synthesized through modification of the hydroxyl groups into carboxy-terminated Pluronic^42^ and coupling to DA, followed by structural characterization, using ^1^H NMR and FT-IR (Scheme S1 and Fig. S1–S5, ESI†). Subsequent copolymerization of F127DA and DA in mild reaction conditions, using different ratios of water and ethanol with Tris-base, yielded F127@PDA NPs. Based on the reaction conditions spherical NPs were obtained in a range from 40 ± 5.4 to 100 ± 14.9 nm (Fig. 2, Fig. S6 and Table S1, ESI†) with a zeta potential from −4 to −19 mV, which is similar to the zeta potential of F127 micelles^43^ and ultrasmall PEG-polydopamine NPs.^44^ Those findings indicate the successful incorporation of Pluronic on the surface of F127@PDA NPs as the zeta potential of bare PDA was found to be approximately −40 mV.^45^

During the synthesis it was noted that the NP size depends both on the ratio of the starting materials and the amount of added ethanol. To further elucidate this observation, *in situ* particle formation was studied in presence of different ethanol amounts by measuring the hydrodynamic diameter and absorbance over time (Fig. 2c and Fig. S7, ESI†). Evaluation of the hydrodynamic diameter over time (Fig. 2c), showed that the NP formation occurs within an initial fast growth phase, followed by slower growth. The seeds formed in presence of 10% EtOH (44.5 nm) are smaller compared to 35% EtOH (62.9 nm) and the inflection point is shifted from 3 to 7 h by increasing the EtOH content. Since the polymerization of polydopamine is characterized by colour change from colourless to dark brown,^36,46,47^ absorbance in the visible range (400–700 nm) was monitored over time (Fig. S8, ESI†) confirming slower polymerization with a higher EtOH ratio. The impact of EtOH on the nucleation and growth kinetics of F127@PDA is in line with reported data on PDA NP formation.^48,49^

As larger NPs (>100 nm) have shown poor penetration and accumulation in solid tumours, such as pancreatic cancer,^50^ we have prepared 40 nm (F127@PDA_40), 60 nm (F127@PDA_60) and 100 nm (F127@PDA_100) nanocarriers for further evaluation trough copolymerization of DA and F127DA (20 : 1 molar ratio) by varying the amount of ethanol (10%, 20% and 35%, respectively). Following the synthesis, the stability of the nanocarriers and the formation of protein corona were explored, since they can change the physicochemical properties altering cell uptake.^51,52^ The colloidal stability of F127@PDA_40, F127@PDA_60 and F127@PDA_100 was assessed in physiological conditions (phosphate-buffer saline, PBS pH 5.5–8.5) and culture media containing serum proteins (DMEM with 0–10% FBS) (Fig. S9 and Table S2, ESI†). No significant changes in size and absorbance spectra were observed after 72 h incubation at 37 °C. Additionally, long-term storage of F127@PDA NPs was evaluated trough lyophilization. The carriers formed a brown solid, which could be readily re-dispersed in water without altering the hydrodynamic size significantly (Table S2, ESI†).

After successful synthesis and characterization, post-synthetic modification of F127@PDA NPs was explored trough fluorescent labelling. To render fluorescent nanocarriers, amino-functionalized fluorescein (Scheme S2, ESI†) was attached *via* Michael addition due to the presence of quinones and indoles in the polydopamine backbone. The successful modification was validated through detection of the characteristic fluorescein peak in the absorbance and fluorescence spectra (Fig. S10, ESI†).

The loading content (w/w) of the fluorescein derivative was determined by UV-Vis and was found to be 9% for F127@PDA@_40 NPs and 6% for F127@PDA@Fl_60 and F127@PDA@Fl_100. No significant changes in size and zeta-potential were observed after the modification compared to F127@PDA NPs (Table S3, ESI†). It should be noted that this post-modification strategy can be employed to attach various (bio)molecular species containing thiol or amino groups as it was demonstrated by immobilization of peptides^53^ and antibodies^54^ to polydopamine.

### Cytotoxicity of F127@PDA NPs

**Fig. 3:**
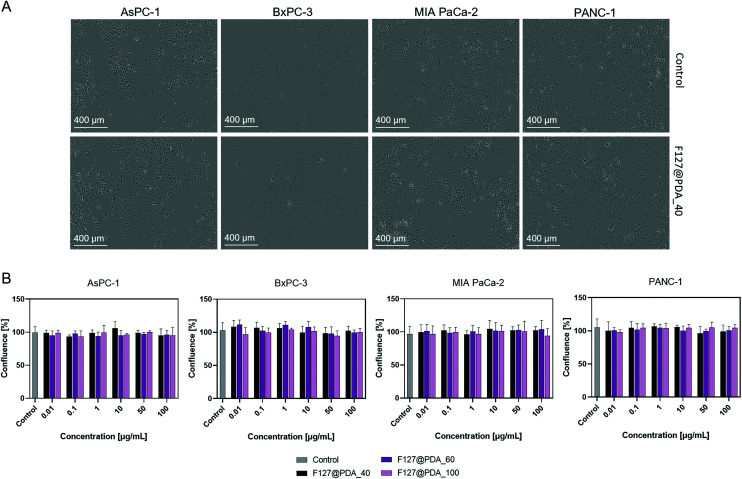
*In vitro* toxicity of F127@PDA NPs over 72 h. Brightfield images of different PDAC control cells and cells treated with 100 µg mL^−1^F127@PDA_40 for 72 h (A). *In vitro* cytotoxicity of F127@PDA_40, F127@PDA_60 and F127@PDA_100 in AsPC-1, BxPC-3, MIA PaCa-2 and PANC-1 cell lines after 72 h incubation determined by live cell analysis. Data are expressed as mean ± SD obtained from three separate measurements (B).

Pancreatic cancer is characterized with high morphological heterogeneity, which leads to poor drug-response.^55–57^ This heterogeneity needs to be taken into account during the study of cell uptake and toxicity to design efficient drug delivery systems (Fig. S11, ESI†). For instance, more than 95% of pancreatic cancers carry *KRAS*^*G12D*^ mutations and 70% *TP53* mutations.^58^ Therefore, *in vitro* cytotoxicity of F127@PDA_40, F127@PDA_60 and F127@PDA_100 has been assessed using four morphologically and genetically different pancreatic cancer cell lines, AsPC-1 (*KRAS*^*G12D*^, *TP53*^C135fs*35^), BxPC-3(*wt-KRAS*, *TP53*^*Y220C*^), MIA PaCa-2(*KRAS*^*G12C*^, *TP53*^*R248W*^) and PANC-1 (*KRAS*^*G12D*^, *TP53*^*R273H*^) over a range of concentrations (0.01–100 µg ml^−1^). Cell growth of different cell lines treated with F127@PDA NPs was monitored by real time *in vitro* micro-imaging using the IncuCyte System (Sartorius, Germany) and MTS endpoint assay for 72 h. The IncuCyte system allows real time-point monitoring of cell growth by determining the confluence of the cells and displaying the morphological changes associated with the treatment (Videos S1 and S2, ESI†). As shown in Fig. 3, none of the cell lines showed changes in morphology or density even when incubated with the highest concentration (100 µg mL^−1^) of NPs over 72 h.

Live cell analysis allows for quantification of the confluence percentage as a function of time, a value which is directly linked to the density of cells. The main advantage of live-imaging systems, compared to the common endpoint assays, such as MTS, nuclei count or CellTiter glow, is that it enables comparison between different time points and normalization of the data obtained in the same well over time.^59^ In addition, the built-in software quantifies the cell surface area coverage as confluency values, so that it is possible to express the cell growth as a ratio between endpoint and time zero, eliminating possible errors in cell seeding and interactions of the NPs with the colorimetric reagent. The analysis confirms that the F127@PDA NPs show no significant difference compared to the control cells. Additionally, viability was also evaluated using widely employed MTS proliferation assay, confirming the results obtained by live cell imaging showing that the nanocarriers have no significant impact on the viability of the cells over the studied concentration range for 72 h (Fig. S12, ESI†).

### Cell internalisation of F127@PDA NPs in different cell types

The cell internalization of nanocarriers depends on their interactions with the cell membrane, which is generally followed by endocytosis.^60^ Various factors, such as the physicochemical and mechanical properties of the nanocarriers, as well as differences in cellular properties such as metabolic status, membrane protein expression and active trafficking pathways, influence the cellular uptake.^61,62^

To undersand the cell uptake of the described nanocarrier systems, flow cytometry and confocal studies were performed using fluorescein-labelled F127@PDA@Fl_40, F127@PDA@Fl_60 and F127@PDA@Fl_100 nanocarriers in the four pancreatic cancer cell lines (AsPC-1, BxPC-3, MIA PaCa-2 and PANC-1). For both the internalisation and intracellular localisation studies, NPs were administrated for 24 h to minimize the proliferation effect that might result in dilution of intracellular NPs, since the doubling time of most mammalian cells is longer than 24 h.^63^

**Fig. 4:**
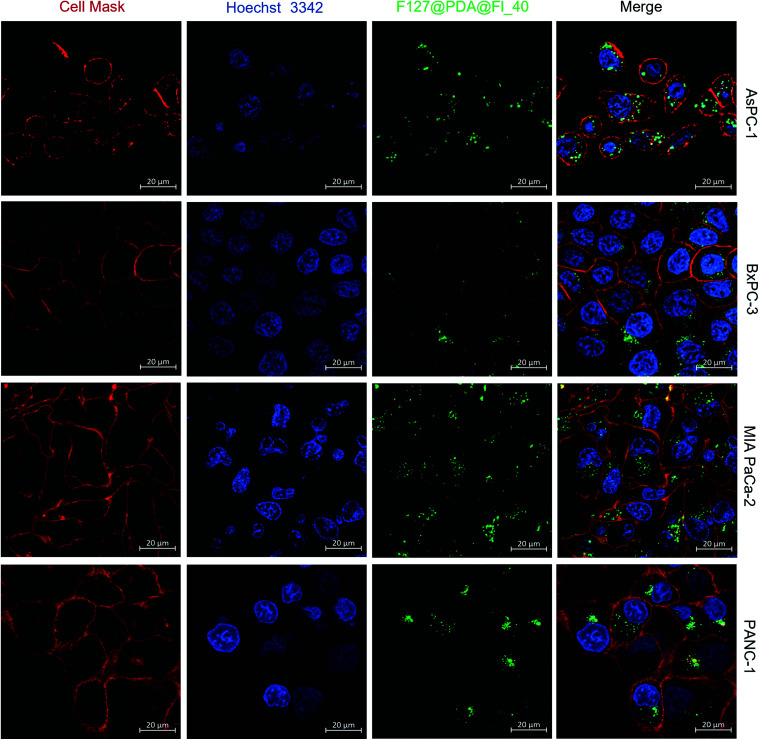
Intracellular localisation of F127@PDA NPs. Fluorescent images of PDAC cells (AsPC-1, BxPC-3, MIA PaCa-2 and PANC-1) after 24 h incubation with 50 μg mL^−1^F127@PDA@Fl_40 NPs acquired with confocal microscopy. Cells were incubated with CellMask (deep red) and Hoechst 33342 (blue) to stain cell membrane and nuclei, respectively. Green channel captured the fluorescence of NPs.

Cell membranes were labelled to differentiate intracellular NPs from those adhered to the cell surface. As shown by confocal microscopy images (Fig. 4), the NPs accumulate in the perinuclear region rather than being scattered throughout the cytoplasm. 3D images (z-stack) of the cells provide additional confirmation that the particles are internalised within the cells (Fig. S13 and Videos S3–S6, ESI†). However, a large number of NPs accumulated on the surface of PANC-1 cells, which was not observed for the other cell lines.

**Fig. 5:**
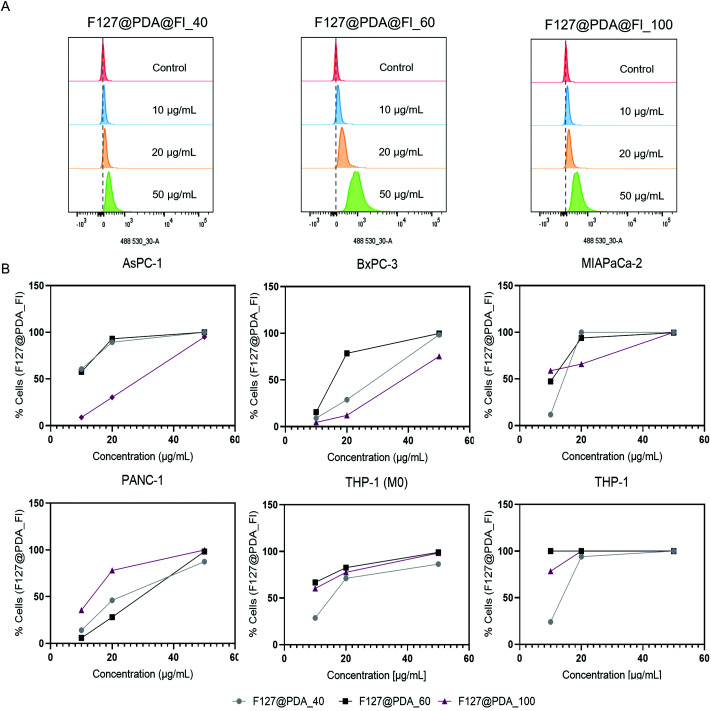
Quantification of F127@PDA uptake using flow cytometry. Cells containing fluorescent NPs is assigned by cells of higher fluorescence intensity than the threshold intensity (dotted grey vertical line) of the untreated samples (A). Percentage of cells containing F127@PDA@Fl NPs in different cell types incubated with different concentrations of NPs obtained from the forward scattering (FSC) vs FITC graph after flow cytometry analysis (B).

The impact of nanocarrier size on the uptake to different PDAC cells was further quantified using a well-defined flow cytometry method previously reported by Shin *et al.*^63^ First, the influence on the side scattering of the cells was measured with increasing F127@PDA@Fl concentration as shown for BxPC-3 cells in Fig. S14, ESI.† No difference in the side scattering was observed after NP treatment for all selected cell lines (data not shown). Following this initial assessment, the fluorescein channel was used for quantification, and it was observed that the fluorescence distributions of the fluorescein channel shift to higher intensities with increasing concentrations of NPs (Fig. 5a), consistent with a higher number of NPs in the cells. The uptake of F127@PDA@Fl increases in a dose-dependent manner within all PDAC cell lines (Fig. 5b). Twenty µg mL^−1^ of the smaller (40 and 60 nm) NPs was sufficient to achieve cell uptake within more than 90% of the AsPC-1 and MIA PaCa-2 cells, while concentrations above 50 µg mL^−1^ were required to achieve the same uptake percentage within BxPC-3 and PANC-1 cells. For larger NPs (100 nm), 90% target loading in all PDAC cells was achieved above 50 µg mL^−1^ except PANC-1 for which 20 µg mL^−1^ were sufficient. Differences in the cell uptake of the four PDAC cell lines were observed, with all cells but PANC-1 preferentially taking up smaller nanoparticles at lower concentrations. This observation clearly points out that it is important to include different cancer cell types into the studies, rather than using one cell line for nanocarrier *in vitro* validation.

In addition to the different PDAC cell lines, the NP uptake into monocytic-like THP-1 cells and THP-1 differentiated macrophages (M0) was also explored, as they were employed to assess the immunocompatibilty of the drug delivery system. A significantly higher uptake level was noted in THP-1 cells, where the uptake was almost complete at lower concentrations (10 µg mL^−1^), apart from the 40 nm particles. Similar results were previously reported for the cell uptake of TiO_2_ ^63^ and polystyrene^64^ NPs, concluding that THP-1 cells take up NPs much faster using. It should be noted that unlike other studied cells which are adherent, monocytic THP-1 cells are the only suspension cell line used to study F127@PDA NP uptake.

In addition to the percentage of fluorescent cells, the median fluorescence intensity (MFI) for individual cells was determined and normalized to the background level of each cell line.^65^ The MFI of suspension THP-1 cells is significantly higher compared to THP-1 (M0) and PDAC cells, which are adherent, (Fig. S14, ESI†). The fluorescence intensity for 40 nm NPs was also significantly higher in AsPC-1 and MIAPaCa-2 cells compared to BxPC-3, Panc-1 and THP-1 (M0) Taking all of the data into account F127@PDA_40 showed a higher level of overall uptake by PDAC cells compared to THP-1 and THP-1 M(0) and was therefore used for subsequent drug release studies.

### Immunocompatibility and interactions with monocytes and differentiated macrophages

After intravenous administration, nanocarriers interact with different blood components, and interactions with the immune system as well as the clearance by the reticuloendothelial system are thought to be the main reason for the observed low levels of NPs at the tumour site.^66,67^ The modulation of the immune system can cause mild adverse reactions but also fatal immune complications. Despite these observations, the assessment of immunocompatibility is often disregarded at early stages of development.^68^ However, *in vitro* evaluation of interactions with the immune system are relevant to determine the dose-range for *in vivo* studies and assess the safety and tolerance of the carrier.

Monocytic THP-1 and differentiated THP-1 (M0) macrophages were used as cellular models to evaluate immunocompatibility.^69^ The cell uptake studies showed that both THP-1 and THP-1 (M0) take up F127@PDA NPs with no significant effect on the viability (Fig. S15, ESI†). Since monocytic THP-1 cells are suspension cells and post differentiation to THP-1 (M0) cells no longer proliferate, viability was not determined *via* live cell imaging.

**Fig. 6:**
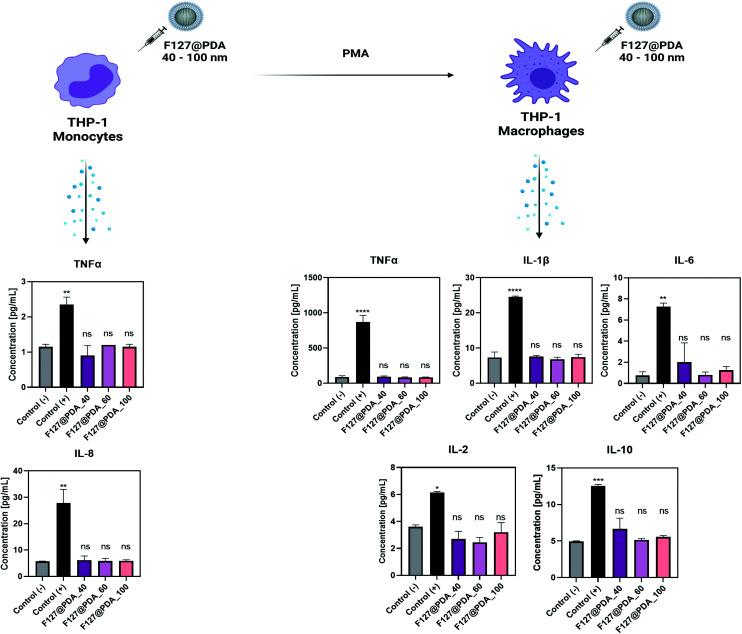
Cytokine profile of THP-1 and THP-1 M(0) cells after 24 h incubation with 10 μg mL^−1^F127@PDA_40, F127@PDA_60 and F127@PDA_100 NPs. LPS is used as (+) control. Data is expressed as mean ± SD. Two-way ANOVA was used for statistical analysis. Significance levels are defined as the following: ns for *p* > 0.05, * for *p* ≤ 0.05, ** for *p* ≤ 0.01, *** for *p* < 0.001, and **** for *p* < 0.0001 compared to the Control (−).

Cytokine profiling was conducted to determine whether F127@PDA NPs induce inflammation. Cytokines are proteins released by immune cells and are accepted as markers for the evaluation of immunotoxicity or pro-inflammatory status.^70^ The concentrations of both proinflammatory (IL-1β, IL-2, IL-6, IL-8, TNF-α, IFN-γ) and anti-inflammatory (IL-4, IL-10, IL-12p70, IL-13) cytokines were determined to evaluate the immune effect of F127@PDA NPs (Fig. 6). The endotoxin lipopolysaccharide (LPS) was used as a positive control to assess the immune response as it significantly increases expression of TNF-α and IL-8 in THP-1 cells and IL-1β, IL-2, IL-6, IL-10 and TNF-α expression by THP-1 (M0) cells.^71^ No significant differences were observed in the cytokine expression after incubation with F127@PDA nanocarriers of different sizes (40, 60 and 100 nm), confirming their immunocompatibility. Further *in vivo* studies will be carried out to fully elucidate the biodistribution and immunocompatibilty of nanocarriers.

### Drug loading and release

Current treatment options for PDAC include gemcitabine, a combination of gemcitabine with nab-paclitaxel (Abraxane) or FOLFIRINOX treatment (5-fluorouracil, leucovorin, irinotecan, and oxaliplatin) for fitter and younger patients.^72^ All four pancreatic cancer cell lines (AsPC-1, BxPC-3, MIA PaCa-2 and PANC-1) show different sensitivity towards the standard of care drugs (Fig. S11, ESI†) making them a suitable *in vitro* model to assess the drug delivery potential of nanocarriers. The delivery of SN38, the active metabolite of irinotecan used in the FOLFIRNOX treatment was evaluated. The metabolic conversion of irinotecan to SN38 occurs in the liver through carboxylesterase 2 cleavage (CES-2), however the CES-2 expression can significantly vary from patient to patient.^73,74^ Both SN38 and irinotecan can undergo lactone ring opening and form a less potent carboxylate form under physiological and basic conditions.^75^ Although SN38 is 1000-fold more potent than irinotecan,^76^ its clinical use is hindered due to the poor solubility and stability, making it an excellent candidate for nanocarrier delivery.^32,77–79^

Based on cell uptake data, 40 nm F127@PDA were used to evaluate SN38 delivery. A loading content of 13.1 ± 3.5% (w/w) was obtained by mixing a suspension of SN38 and F127@PDA_40 NPs, as verified by UV-Vis spectroscopy (Fig. S16, ESI†). Due to the presence of aromatic groups in the polydopamine structure, small molecules can easily be absorbed through π–π stacking or hydrogen bonding.^46,80^ To determine whether the lactone or carboxylesterase form of SN38 was loaded into the carrier, the supernatant was analysed using HPLC^33^ and showed the characteristic peak for the lactone at 2.7 min, indicating the loading of the active SN38 form.

Prior to evaluating the *in vitro* effect of SN38@F127@PDA_40 on PDAC cell lines, release behaviour in PBS (1×, pH 7.4) was determined (Fig. S16, ESI†) showing no burst release and overall release ∼30% after 72 h. However, it should be noted that the poor solubility of SN38 limits precise assessment and quantification of the release profile in physiological conditions.

**Fig. 7:**
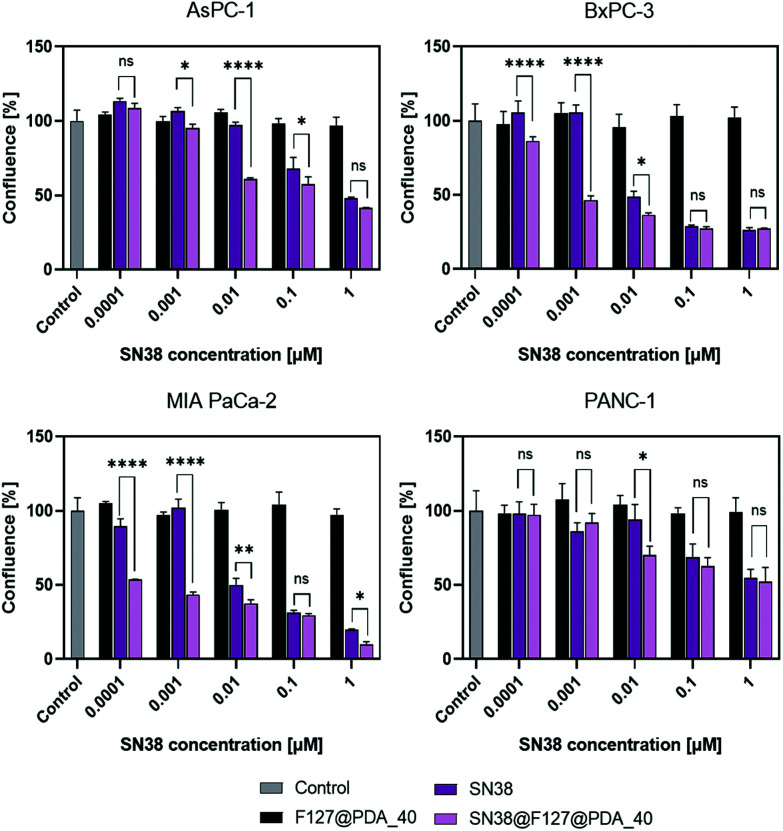
Cytotoxicity of SN38 and SN38@F127@PDA and F127@PDA NPs determined by live cell imaging after 72 h treatment. Data is expressed as mean ± SD from three separate experiments. Two-way ANOVA was used to compare SN38 and SN38@F127@PDA. Significance levels are defined as the following: ns for *p* > 0.05, * for *p* ≤ 0.05, ** for *p* ≤ 0.01, *** for *p* < 0.001, and **** for *p* < 0.0001.

The antiproliferative effect of SN38@F127@PDA_40 was assessed and compared to that of the free SN38 using live cell imaging. AsPC-1, BxPC-3, MIA PaCa-2 and PANC-1 cells were treated for 72 hours making sure the SN38 concentration is the same in both systems. As shown in Fig. 7 and Fig. S17† at higher concentrations (1000 and 100 nM) SN38 loaded F127@PDA_40 NPs have a similar effect as free SN38. However, at concentrations lower than their IC_50_ values (10 nM for AsPC-1 and PANC-1; 1 nM for BxPC-3 and MIA PaCa-2), a significant antiproliferative effect of the SN38 loaded F127@PDA_40 was observed considerably altering the growth curves over time (Fig. S17 and Videos S7, S8, ESI†).

It should be noted that both AsPC-1 and PANC-1 are SN38-resistant cell lines. Yet, a significant effect of SN38@F127@PDA on all PDAC cells was observed. Compared to AsPC-1, BxPC-3 and MIA PaCa-2, PANC-1 cells are least responsive to the SN38 loaded NPs. This is in line with the cell uptake profile of F127@PDA_40 (Fig. 5), which showed lower cell uptake into PANC-1 cells compared to the other cell lines. Hence, other strategies to improve uptake into PANC-1 cells, such as the use of different Pluronic-containing polymers or targeting moieties, need to be considered.

In addition to the proliferation studies, MTS assay was conducted (Fig. S18, ESI†) after treatment with SN38 and SN38@F127@PDA for 72 h and IC_50_ values were calculated from the obtained dose response (Table S4, ESI†). The obtained IC_50_ values for SN38 calculated from the MTS assay correspond to literature reports^81^ and SN38@F127@PDA NPs show lower IC_50_ values then SN38 for all tested cell lines. The IC_50_ values obtained from the live cell imaging were lower than from MTS both for SN38 and SN38@F127@PDA. Similar findings were also noted for several other drugs using real-time cell analysis,^82–84^ which shows the importance to use different methods when determine the effect of new therapies to avoid generating misleading results.

## Conclusions

In summary, we have designed a novel size-tuneable and easily modifiable drug delivery system composed of Pluronic F127 and polydopamine and assessed its potential for drug delivery of hard-to-treat pancreatic cancer. This study highlights several assays for *in vitro* assessment, which consider the heterogeneity of PDAC, and can be used as a tool to gain better understanding of drug delivery systems based on the disease characteristics at an early stage. The F127@PDA carriers were prepared in different sizes (40, 60 and 100 nm) without altering the composition, allowing for the distinct assessment of the size-effect. Incorporation of Pluronic within the structure, rather than adding it to the surface, allowed us to prepare nanocarriers that are stable over prolonged time with a reproducible synthesis. Mechanistic studies showed that the size control can be achieved by increasing the ratio of EtOH in the reaction mixture, which slows down the polymerization. Nanocarriers showed excellent colloidal stability and high loading capacity for hydrophobic and labile irinotecan active metabolite SN38. Viability, cellular uptake and cytokine profile studies of differently sized nanocarriers (40, 60 and 100 nm) demonstrated high bio- and immunocompatiblity for all studied sizes. However, cell uptake into the different PDAC cells, monocytes and macrophages showed variability based on NP size. These findings indicate the importance of exploring different cell types present within the tumour to address the heterogeneity. Finally, SN38 loaded F127@PDA nanocarriers demonstrated a more pronounced effect on proliferation of all cell lines compared to the free drug. Although these initial studies indicated significant difference in the uptake of different carrier sizes, further studies that take into account the diffusion through 3D tumour space need to be performed using relevant organoid and *in vivo* models.

## Author contributions

A.B.P, F.M., E.L. and L.F. contributed equally. L.F. conceptualized the project, and A.B.P., F.M. and E.L performed research experiments. I.A. and B.P. helped with the chemical synthesis. Immunomodulation studies were conducted and analysed with help from H.R. C.O.F contributed to the nanoparticle synthesis and characterization. C.G.L. helped with the flow cytometry analysis. F.R., D.M.E., D.J. and D.F.J. helped with critical comments throughout the project, drafting, and revising the manuscript.

## Conflicts of interest

There are no conflicts to declare.

## Supplementary Material

NR-014-D2NR00864E-s001

NR-014-D2NR00864E-s002

NR-014-D2NR00864E-s003

NR-014-D2NR00864E-s004

NR-014-D2NR00864E-s005

NR-014-D2NR00864E-s006

NR-014-D2NR00864E-s007

NR-014-D2NR00864E-s008

NR-014-D2NR00864E-s009
