# Supplementary material for: Size-tuneable and immunocompatible polymer nanocarriers for drug delivery in pancreatic cancer
Source: Nanoscale. 2022 Apr 19;14(17):6656–69. doi: 10.1039/d2nr00864e (PMC9070568; doi:10.1039/d2nr00864e)
Supplement: NR-014-D2NR00864E-s001 [file NR-014-D2NR00864E-s001.pdf]

Electronic Supplementary Material (ESI) for Nanoscale.
This journal is © The Royal Society of Chemistry 2022

Electronic Supporting Information

Size-tuneable and Immunocompatible Polymer Nanocarriers for Drug Delivery in Pancreatic Cancer

*Andrea Bistrović Popov[a]‡, Francesca Melle[b]‡, Emily Linnane[b]‡, Cristina González-López[a,c], Ishtiaq Ahmed[a], Badri Parshad[a], Christoph O. Franck[a], Hassan Rahmoune[d], Frances M. Richards[e,f], Daniel Muñoz-Espín[c], Duncan I. Jodrell[e,g], David Fairen-Jimenez[b] and Ljiljana Fruk[a]**

[a]BioNano Engineering Lab, Department of Chemical Engineering and Biotechnology, University of Cambridge, Philippa Fawcett Drive, Cambridge CB3 0AS, UK
[b]The Adsorption & Advanced Materials Laboratory (A^2ML), Department of Chemical Engineering and Biotechnology, University of Cambridge, Philippa Fawcett Drive, Cambridge CB3 0AS, UK
[c]CRUK Cambridge Centre Early Detection Program, Department of Oncology, Hutchison/MRC Research Centre, University of Cambridge, Cambridge CB2 0RE, UK
[d]Department of Chemical Engineering and Biotechnology, University of Cambridge, Philippa Fawcett Drive, Cambridge CB3 0AS, UK The affiliation should be the institution where the work was conducted.
[e]Cancer Research UK Cambridge Institute, University of Cambridge, Li Ka Shing Centre, Cambridge CB2 0RE, UK
[f]Translational Medicine, Oncology R&D, Astra Zeneca, Cambridge CB4 0WG, UK
[g]Department of Oncology, University of Cambridge, Hutchison/MRC Research Centre, Cambridge Biomedical Campus, Cambridge, CB2 0XZ, UK

[*]Corresponding author. E-mail: lf389@cam.ac.uk
‡Contributed equally to this paper.

Content	Page
1. Materials and methods	2
1.1. Characterization techniques	2
1.2. Synthesis of Pluronic-dopamine monomer (**F127DA**)	3
1.3. Synthesis and characterization of **F127@PDA** NPs	7
1.4. Colloidal stability of **F127@PDA** NPs	10
1.5. Synthesis and characterization of **Fluorescein-TEG-NH_2**	11
1.6. Functionalization of **F127@PDA NPs** with **Fluorescein-TEG-NH_2**	14
2. In vitro evaluation of **F127@PDA** NPs	15
2.1. Cytotoxicity studies	15
2.2. Cell internalization	16
2.3. Immunomodulation studies	19
2.4. Drug loading and release in vitro	20
3. References	22

1. Materials and Methods

All materials were purchased from either Acros Organics (UK), Alfa Aeser (UK), Sigma-Aldrich (UK) or TCI Chemicals (UK) in the highest purity available and used without further purification.

1.1. Characterization techniques

^{1}H measurements were carried out using 400 MHz QNP Cryoprobe Spectrometer (Bruker) by the NMR service of the Department of Chemistry, University of Cambridge. UV-Vis absorption spectra were obtained with an Agilent Cary 300 Spectrophotometer. Fluorescence emission spectra were obtained using a Varian Cary Eclipse Fluorescence Spectrophotometer using excitation and emission splits of 5 nm. DLS and zeta potential measurements were recorded using a Zetasizer Nano Range instrument (Malvern Panalytical). FTIR spectroscopy was carried out using a Bruker Tensor 27 spectrometer with samples pressed into KBr pellets. SEM images were obtained using a FEI Verios 460. Samples were suspended in water and drop cast on lacey carbon copper grids (Agar Scientific).

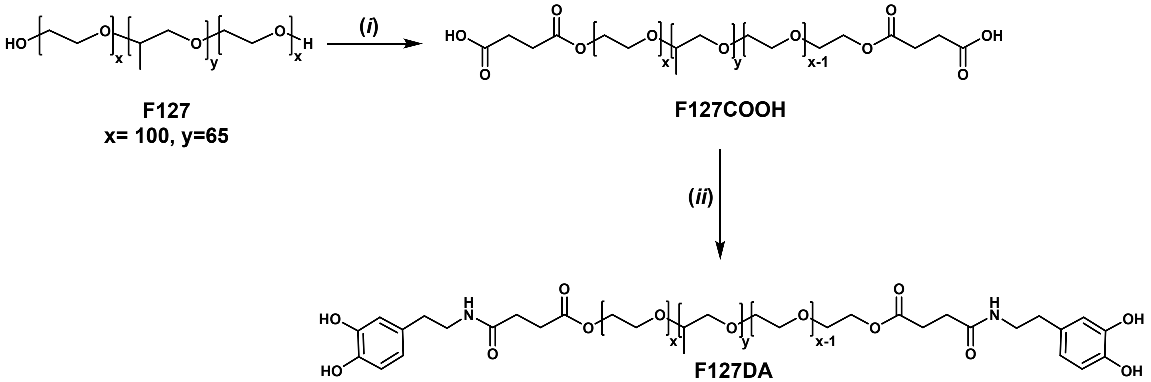

Scheme S1. Reaction conditions: (*i*) succinic anhydride, pyridine, rt, 72 h (*ii*) dopamine hydrochloride, NHS, DMAP, DCC, DMF, rt, 24 h.

1.2. Synthesis of Pluronic-dopamine monomer (F127DA)

Carboxyl-terminated Pluronic (F127COOH). Carboxyl-terminated F127 (F127COOH) was prepared according to the procedure reported by Li et al.[1] F127 (30.0 g, 2.5 mmol) was dissolved in pyridine (60 mL) and succinic anhydride (7.1 g, 71.4 mmol) was added. The reaction mixture was stirred under argon for 72 hours. Subsequently, CH_2Cl_2 (150.0 mL) was added to dilute the reaction mixture and washed with saturated sodium chloride solution three times. The organic layer was dried over anhydrous magnesium sulphate overnight, filtered, and concentrated by rotary evaporation. The residue was precipitated with cold diethyl-ether (31.5 g, yield: 95%). ^{1}H NMR (400 MHz, $CDCl_3$): δ (ppm) 1.42–0.84 (m, 195H, CH_3-a), 2.66–2.51 (m, 8H, CH_2-f,g), 3.45–3.26 (m, 67H, CH-b), 3.55–3.42 (m, 132H, CH_2-c), 3.85–3.54 (m, 833H, CH_2-d), 4.29–4.18 (m, 4H, CH_2-e).

Pluronic-dopamine (F127DA). F127COOH (2.0 g, 0.2 mmol) was dissolved in DMF (25 mL) followed by addition of NHS (60.2 mg, 0.52 mmol), DMAP (2.5 mg, 0.02 mmol), DCC (120.5 mg, 0.58 mmol) and dopamine hydrochloride (65.5 mg, 0.45 mmol). The reaction mixture was stirred under inert atmosphere for 24 hours. The solvent was removed by rotary evaporation and the resulting product was subsequently dissolved in methanol: water (50: 50), dialyzed against methanol: water (50: 50) for 2 days, and then against water for another 2 days. The final product was obtained in the form of a white power after lyophilization of the dialyzed solution (1.95 g, yield: 78%). ^{1}H NMR (400 MHz, $CDCl_3$): δ (ppm) 1.40–0.89 (m, 195H, CH_3-a), 2.44–2.31 (m, 4H, CH_2-e), 2.71–2.52 (m, 12H, CH_2-f,g,i), 3.41–3.29 (m, 67H, CH-b), 3.55–3.42 (m, 132H, CH_2-c), 3.81–3.55 (m, 843H, CH_2-d), 4.22–4.12 (m, 8H, CH_2-e,h), 6.11–5.96 (m, 2H, NH), 6.51 (dd, J=8.0, 1.5, 2H, Ar-H), 6.67 (d, J=1.2, 2H, Ar-H), 6.76 (d, J=8.0, 2H, Ar-H), 8.09–8.02 (m, 4H, OH).

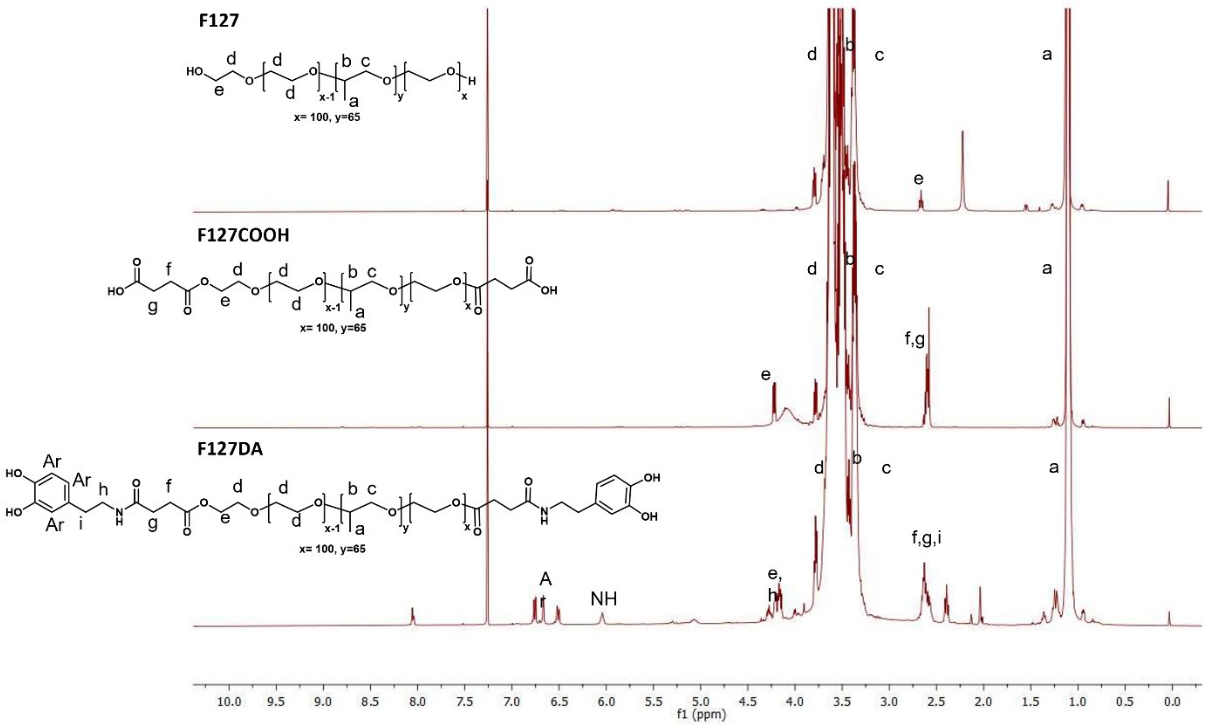

Figure S1. ^{1}H NMR spectra of **F127**, **F127COOH** and **F127DA** in $CDCl_3$.

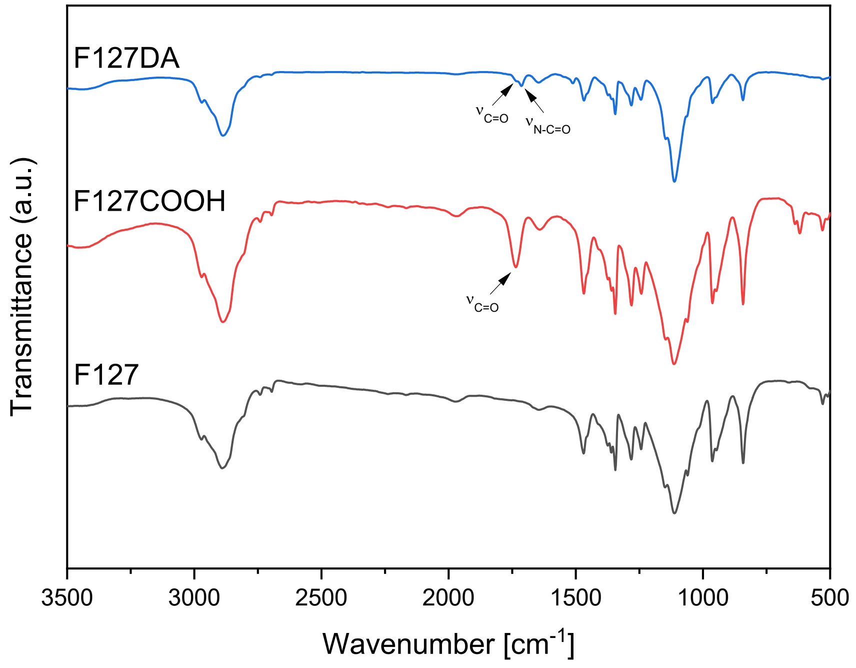

Figure S2. FT-IR spectra obtained for **F127**, **F127COOH** and **F127DA** with KBr pellet.

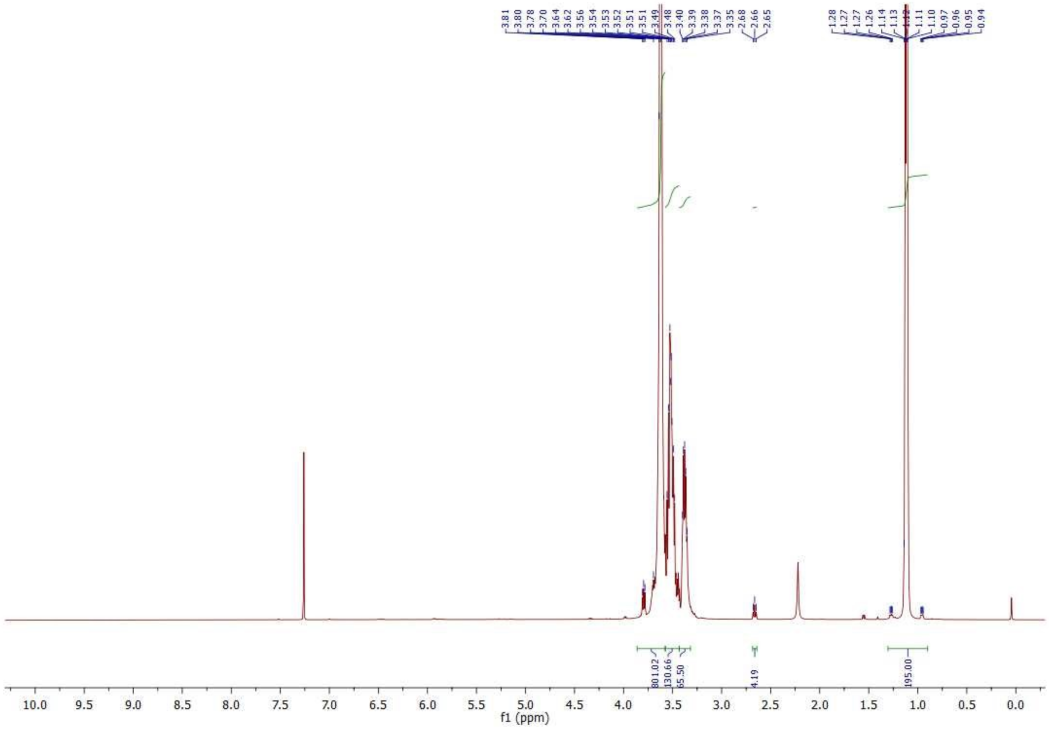

Figure S3. ^{1}H NMR spectra of **F127** in $CDCl_3$.

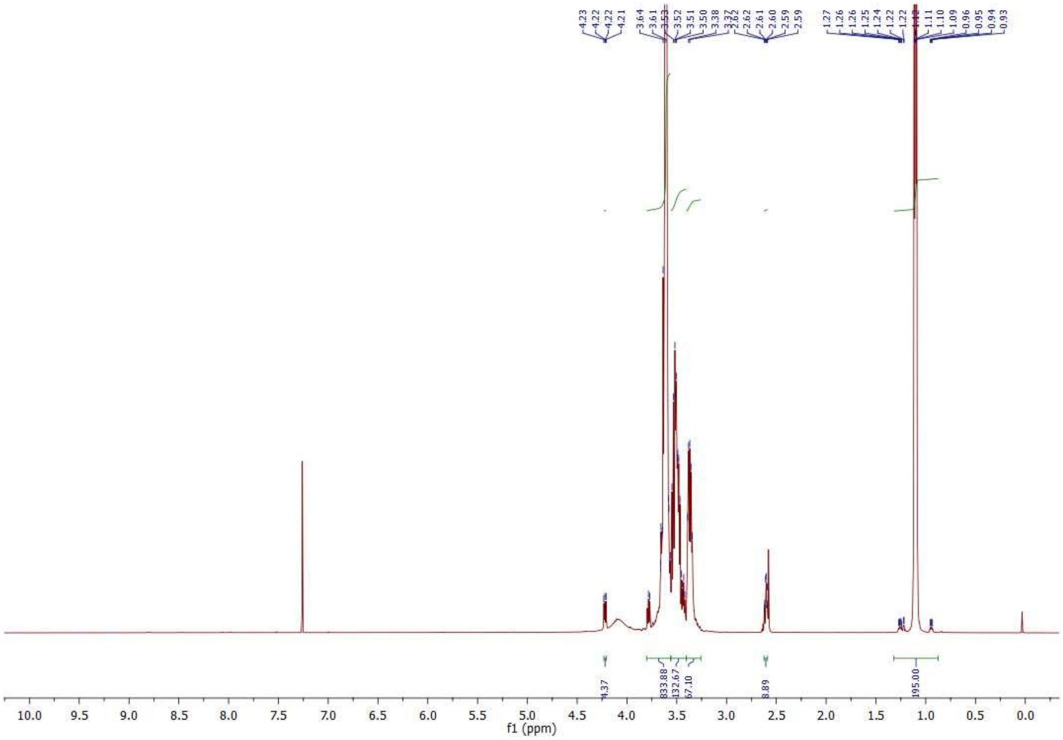

Figure S4. ^{1}H NMR spectra of **F127COOH** in $CDCl_3$.

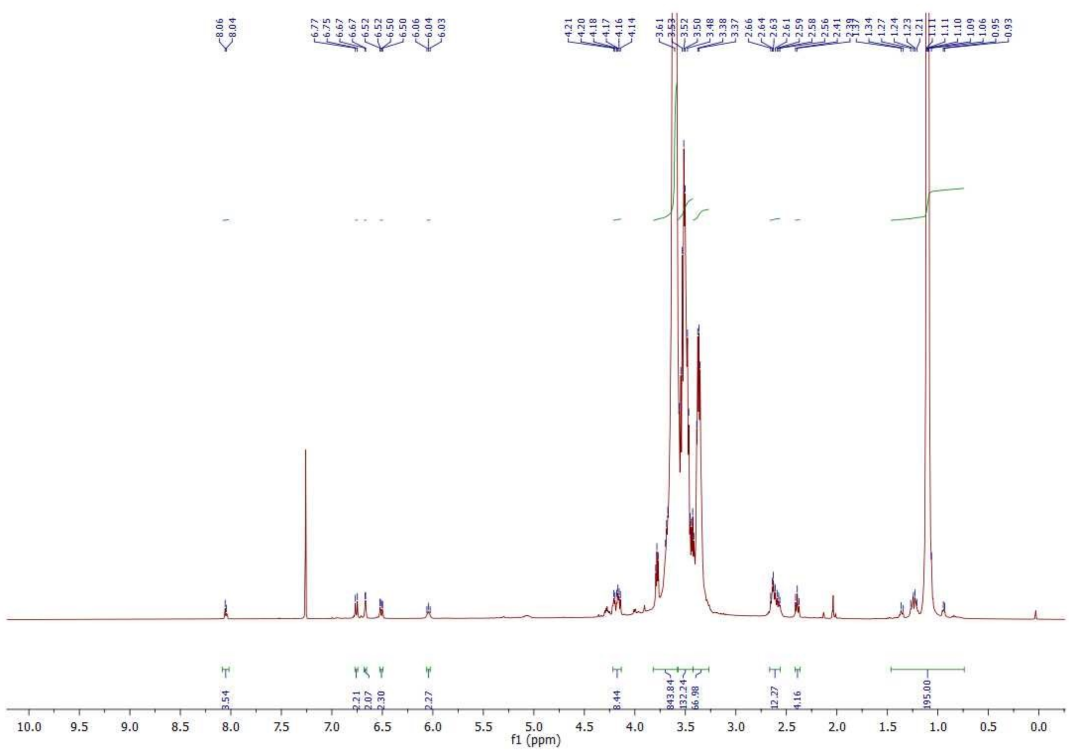

Figure S5. ^{1}H NMR spectra of **F127DA** in $CDCl_3$.

1.3. Synthesis and characterization of F127@PDA NPs

Table S1. DLS and zeta potential (ζ) measurements of **F127@PDA** samples. Errors are the standard deviation of the triplicate data. Sizes from STEM images were determined from the mean of >100 measurements of spherical particles, with the associated error being the standard deviation.

n(DA): n(F127DA)	m(DA) [mg]	m(F127DA) [mg]	V(EtOH) [mL]	V(H_2O [mL]	Hydrodynamic diameter [nm]	PDI	STEM size [nm]
20:1	13.7	45.7	3	27	59.2±1.3	0.060	45.9±5.4
20:1	13.7	45.7	6	24	76.6±1.2	0.048	62.5±5.9
20:1	13.7	45.7	9	21	104.4±0.8	0.005	87.8±9.0
20:1	13.7	45.7	10.5	19.5	117.0±1.7	0.047	103.3±14.9
20:1	13.7	45.7	15	15	164.1±4.6	0.021	140.9±15.4
10:1	13.1	91.6	6	24	95.8±2.2	0.027	73.4±7.2
20:1	13.7	45.7	6	24	106.5±1.4	0.032	87±7.8
50:1	13.9	32.5	6	24	120.4±3.5	0.043	99.3±9.7
100:1	14.0	24.5	6	24	141.8±4.2	0.009	110.2±9.9

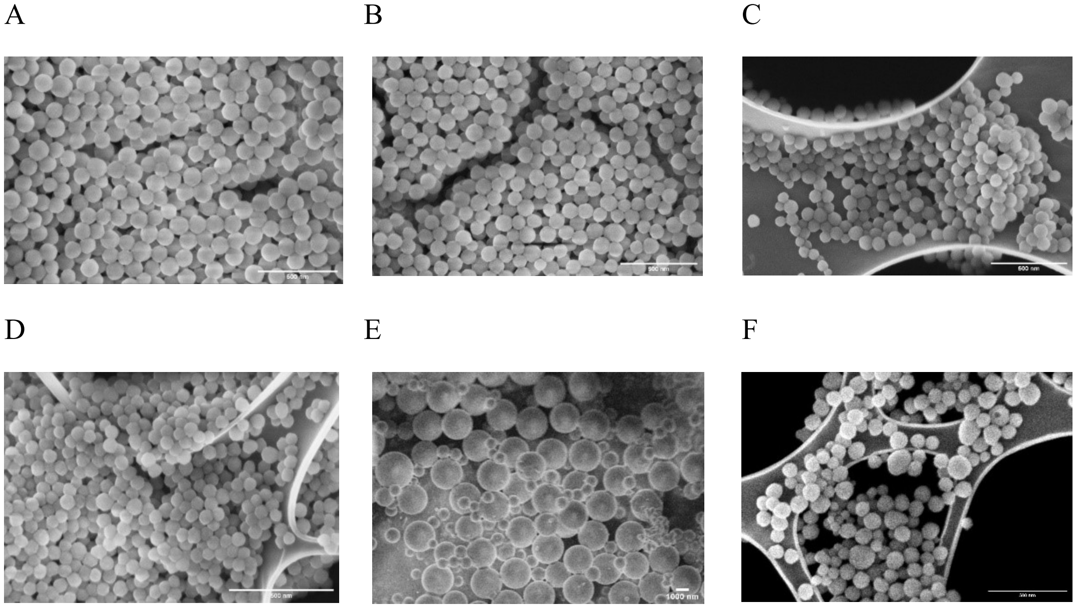

Figure S6. STEM images of **F127@PDA** NPs prepared in a 10:1 (A), 20:1 (B), 50:1 (C), 100:1 (D), 0:1 (D) and 1:0 (E) molar ratio of DA:F127DA with 35% ethanol in the reaction mixture.

A **C**

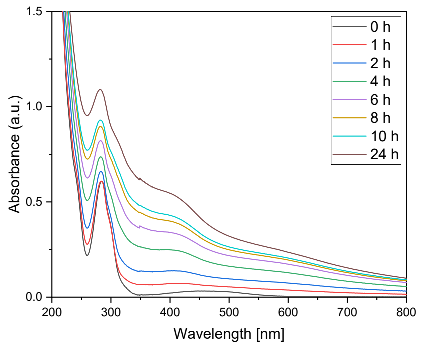

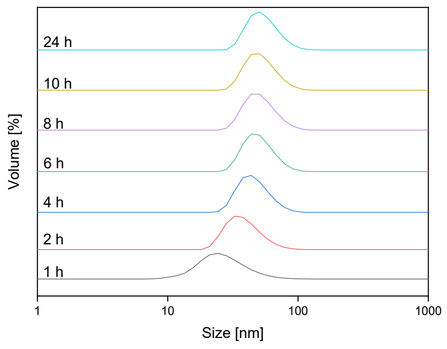

B

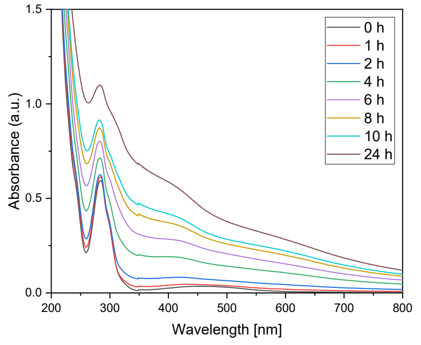

D

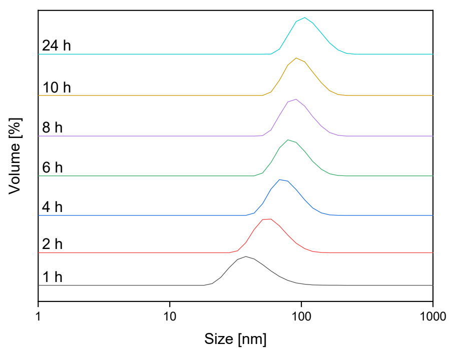

Figure S7. UV-Vis spectra of the reaction mixture during the formation of **F127@PDA_40** (A) and **F127@PDA_100** (B). Time-resolved dynamic light scattering (DLS) monitoring the evolution of hydrodynamic diameter distributions of the reaction suspension at different reaction times for **F127@PDA_40** (C) and **F127@PDA_100** (D).

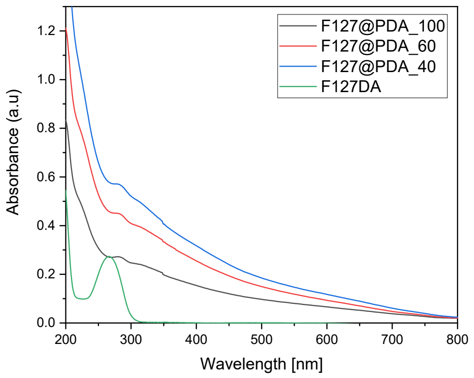

Figure S8. UV-Vis spectra of **F127DA**, **F127@PDA_40**, **F127@PDA_60** and **F127@PDA_100** in water at a concentration of 0.1 mg/mL.

1.4. Colloidal stability of F127@PDA NPs

Table S2. Hydrodynamic diameter and zeta potential (ζ) for **F127@PDA_40**, **F127@PDA_60** and **F127@PDA_100** in water, PBS pH 5.5–8.5 and DMEM with FBS 0–10% after 72 hours incubation at 37ºC. Errors are standard deviations of the triplicate data.

Dispersant	*F127@PDA_40* Z-avg (nm)	PDI	ζ (mV)	*F127@PDA_60* Z-avg (nm)	PDI	ζ (mV)	*F127@PDA_100* Z-avg (nm)	PDI	ζ (mV)
**Water**	61.3±1.5	0.044	-16.5±1.1	83.3±2.2	0.017	-17.3±2.7	116.2±1.9	0.016	-19.0±1.3
**Water[b]**	67.5±2.1	0.111	-14.5±0.4	95.1±2.2	0.095	-15.7±3.2	123.8±1.9	0.105	-16.4±1.7
**PBS 4.5**	64.9±0.9	0.096	-6.7±0.9	85.1±1.7	0.029	-8.4±0.7	119.2±2.2	0.008	-5.3±1.9
**PBS 6.5**	69.9±1.6	0.075	-4.9±0.3	83.8±1.1	0.049	-4.9±1.8	120.4±0.5	0.040	-8.4±1.4
**PBS 7.5**	67.6±1.6	0.066	-5.2±1.7	86.3±0.5	0.050	-5.3±1.2	114.9±2.9	0.035	-6.4±1.7
**PBS 8.5**	70.4±0.7	0.092	-6.1±0.5	84.6±1.3	0.036	-7.7±0.3	126.9±1.3	0.060	-4.7±0.3
**DMEM**	71.4±2.1	0.069	-4.8±0.6	86.4±2.1	0.044	-4.5±0.6	121.6±1.5	0.076	-6.4±0.7
**DMEM 10%FBS**	68.1±4.4	0.296	-5.4±0.2	82.6±5.1	0.160	-6.3±1.9	116.2±3.2	0.139	-5.0±2.1

[b]After lyophilization.

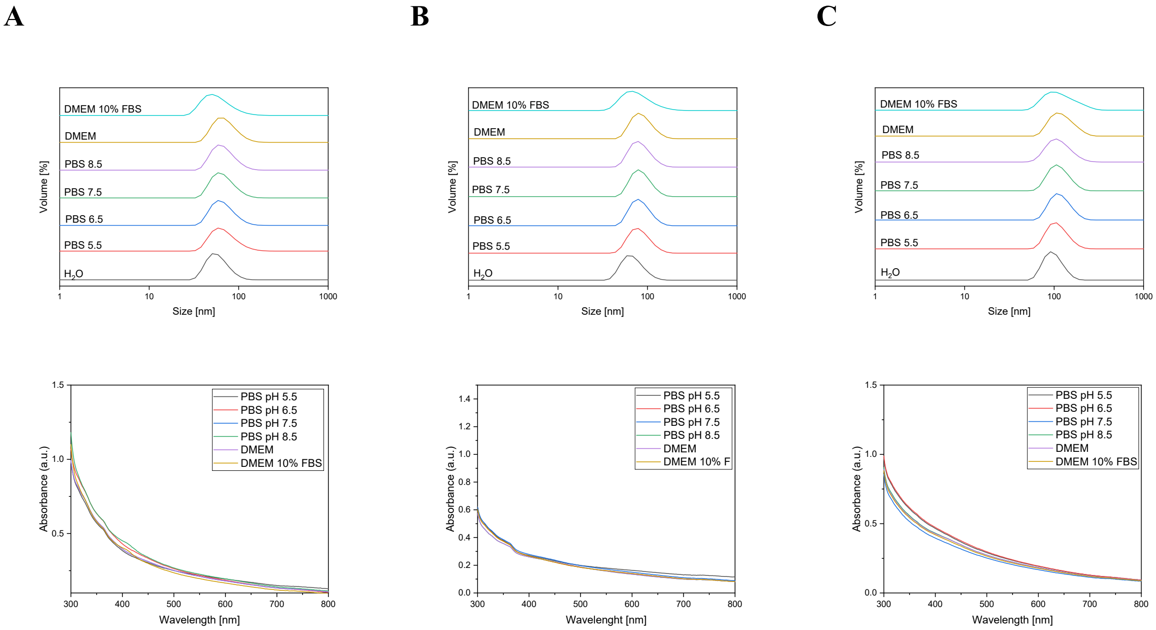

Figure S9. Colloidal stability of **F127@PDA_40** (A), **F127@PDA_60** (B) and **F127@PDA_100** (C) measured using DLS (top) and UV-Vis spectroscopy (bottom).

1.5. Synthesis of Fluorescein-TEG-NH_2

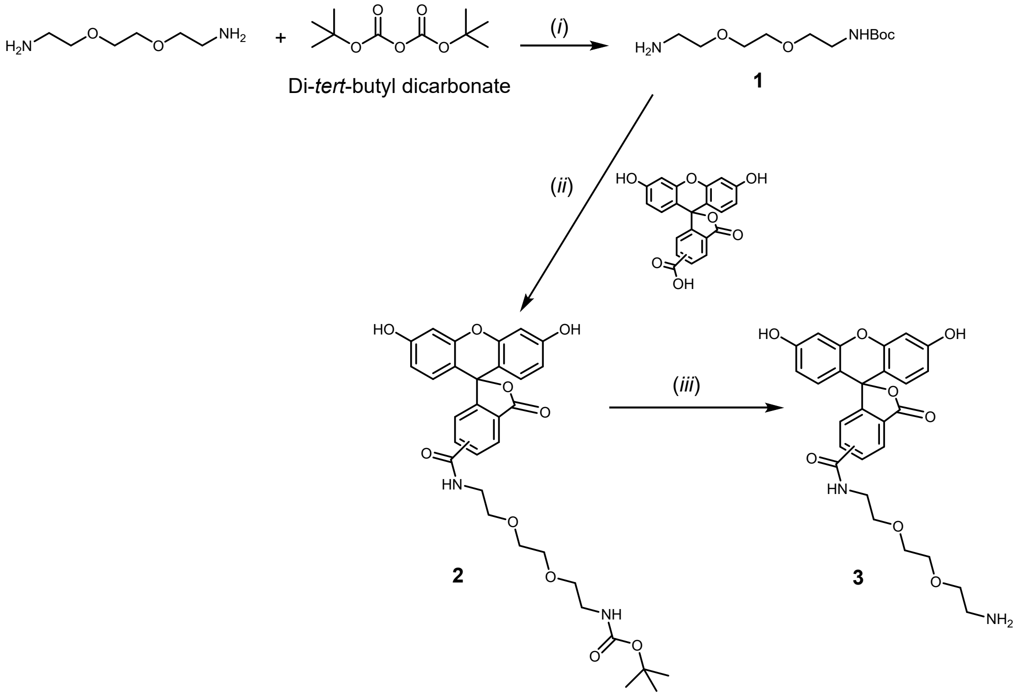

Scheme S2. Reaction conditions: (*i*) DCM, 0 ºC, 6 h; overnight, rt (*ii*) HATU, DIPEA, DMF, overnight, rt (*iii*) TFA, DCM, 3 h, rt.

***N-Boc-2,2'-(ethylene-l,2-dioxy)bisethylamine* (1).** Compound **1** was synthesized according to a reported method with slight modification.[2] A solution of di-*tert*-butyl dicarbonate (11.0 g, 60.0 mmol) in 250 mL CH_2Cl_2 was added dropwise to a solution of 2,2′-(ethylenedioxy)bis(ethylamine) (30.0 mL, 200 mmol) in 200 mL dry CH_2Cl_2 at 0 °C under nitrogen atmosphere over a period of 6 h. The reaction mixture was stirred at 0 °C for 6 h and then at room temperature overnight. The mixture was extracted with 200 mL brine three times and 200 mL water. The organic phase was collected and dried over Na_2SO_4. The solvent was evaporated under vacuum to a give colorless oil (6.1 g, 71%). **^{1}H NMR (400 MHz, $CDCl_3$):** δ (ppm) 1.41 (s, 9H). 2.61 (t, J = 5.3 Hz, 2H), 3.02 (m, 2H), 3.24-3.36 (m, 4H), 3.38-3.44 (m,4H), 5.41 (br, 1H). **HR-MS (ESI):** *m/z* [M^+] calculated for $C_{11}H_{24}N_2O_4$: 248.1713; found: 248.1728.

Tert-butyl (2-(2-(2-acetamidoethoxy)ethoxy)ethyl)carbamate--3',6'-dihydroxy-3*H*-spiro[isobenzofuran-1,9'-xanthen]-3-one (2). 5(6)-carboxyfluorescein (1.0 g, 2.65 mmol) was dissolved in anhydrous DMF (15 mL) under argon and HATU (1.22 g, 3.20 mmol) and DIPEA (1.029 g, 1.4 mL, 7.98 mmol) were added to the solution. The reaction mixture was stirred at room temperature under argon for 30 min. The solution of *N-Boc-2,2'-(ethylene-l,2-dioxy)bisethylamine* (**1**) (0.86 g, 3.45 mmol) in anhydrous DMF (5 mL) was slowly added under Ar. The reaction mixture was stirred overnight at room temperature. The solvent was removed under reduced pressure to obtain dark orange residue. Silica gel column chromatography using CH_2Cl_2:MeOH (9:1) gave pure compound **2** as orange thick oil (1.4 g, 2.31 mmol, 87 % yield). **^{1}H NMR (400 MHz, DMSO-d_6, mixture of isomers): δ (ppm)** 1.35-1.38 (m, 6H), 1.41-1.44 (m, 12H), 3.13 (t, J = 7.1 Hz, 2H), 3.29-3.31 (m, 4H), 3.36-3.38 (m, 2H), 3.42-3.45 (m, 4H), 3.58-3.60 (m, 6H), 3.65-3.71 (m, 6H), 3.72 (bs, 2H), 6.51-6.55 (m, 4H, Ar-H), 6.57-6.59 (m, 2H, Ar-H), 6.70-6.75 (m, 4H, Ar-H),7.24 (d, J = 7.8 Hz, 2H, Ar-H), 7.33(s, 1H, Ar-H), 7.60 (s, 1H, Ar-H), 8.05-8.11 (m, 3H, Ar-H), 8.23 (d, J = 8.1 Hz, 1H, Ar-H), 8.45 (br s, 1H, NH). **^{13}C NMR (100 MHz, DMSO-d_6, mixture of isomers): δ (ppm)** 23.5, 26.7, 31.8, 33.7, 35.2, 36.6, 50.0, 65.5, 74.5, 79.1, 79.3, 82.4, 98.0, 98.1, 104.7, 107.8, 118.0, 118.8, 119.2, 120.3, 122.5, 124.1, 124.4, 128.5, 128.6, 128.7, 130.1, 131.6, 136.0, 147.8, 147.9, 151.6, 151.7, 159.2, 161.7, 164.5. **HR-MS (ESI):** *m/z* [M^+] calculated for $C_{32}H_{34}N_2O_{10}$: 606.2245; found: 606.2214.

***N*-(2-(2-(2-aminoethoxy)ethoxy)ethyl)acetamide--3',6'-dihydroxy-3*H*-spiro[isobenzofuran-1,9'-xanthen]-3-one (3).**To a solution of Boc-protected compound **2** (500 mg, 0.825 mmol) in CH_2Cl_2 (20 mL) was added trifluoroacetic acid (6 mL). The mixture was stirred for 3 hours at room temperature. The solvent was evaporated under reduced pressure to give yellow residue. CH_2Cl_2 (20 mL) was added to the residue and evaporated. This process was

repeated three times (3x20 mL) to remove the trifluoroacetic acid. Toluene (30 mL) was added to the residue and the solvent was evaporated in order to remove the traces of trifluoroacetic acid to give dark orange liquid of compound **3** amine as its trifluoroacetate salt (360 mg, 0.711 mmol, 86 % yield). **^{1}H NMR (400 MHz, DMSO-*d*$_{6}$, mixture of isomers): δ (ppm)** 2.85-2.90 (m, 2H), 2.92-2.96 (m, 2H), 3.05-3.11 (m, 2H), 3.29-3.35 (m, 2H), 3.35-3.45 (m, 2H), 3.47-3.49 (m, 2H), 3.55-3.61 (m, 6H), 3.62-3.65 (m, 6H), 6.49-6.58 (m, 4H, Ar-H), 6.61-6.69 (m, 3H, Ar-H), 7.05-7.10 (m, 2H, Ar-H), 7.19-7.24 (m, 2H, Ar-H), 7.35 (d, *J* = 8.1 Hz, 1H, Ar-H), 7.64 (s, 1H, Ar-H), 7.66 (s, 1H, Ar-H), 8.03-8.16 (m, 3H, Ar-H), 8.23 (d, *J* = 8.1 Hz, 1H, Ar-H), 8.41 (s, 1H). 8.72 (br s, 1H, NH). **^{13}C NMR (100 MHz, DMSO-*d*$_{6}$, mixture of isomers): δ (ppm)** 36.2, 42.2, 54.01, 67.0, 67.1, 68.7, 69.8, 70.0, 70.1, 79.1, 79.2, 102.7, 108.4, 108.5, 113.1, 114.4, 117.3, 120.2, 126.7, 128.6, 129.3, 129.5, 129.7, 135.1, 136.5, 137.8, 152.2, 150.2, 159.0, 160.1, 162.7, 165.0, 165.2, 168.5, 168.6. **HR-MS (ESI):** *m/z* [M^{+}] calculated for $C_{27}H_{26}N_2O_8$: 506.1724; found: 506.1735.

1.6. Fluorescein functionalization of F127@PDA NPs

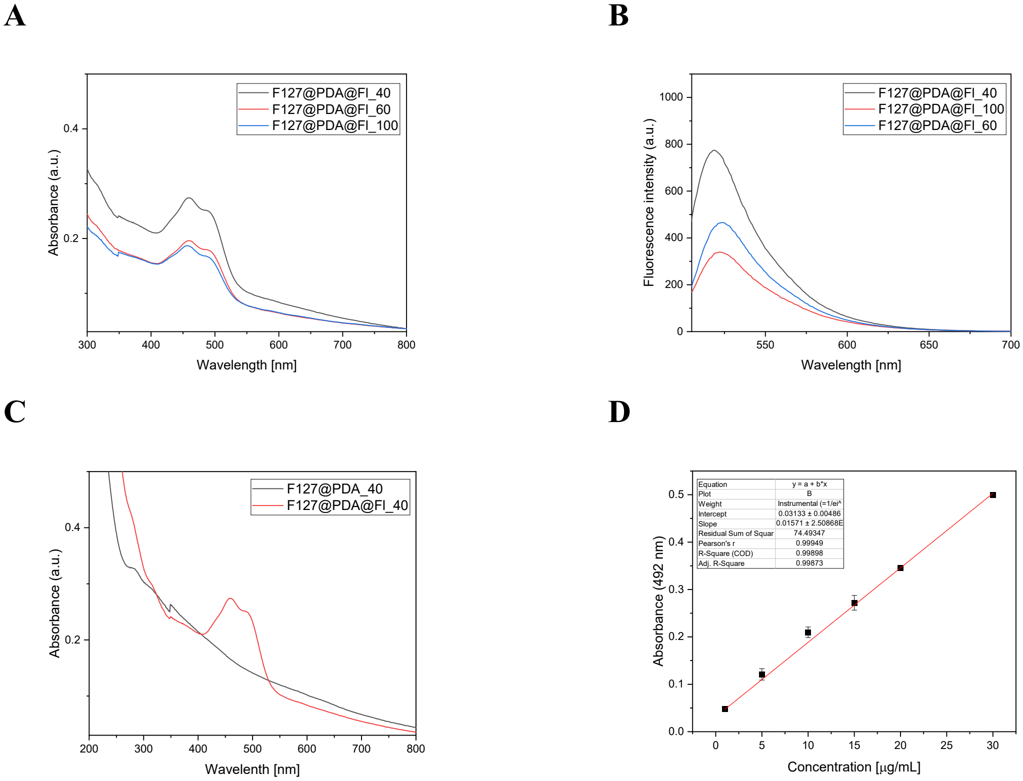

Figure S10. Spectroscopic characterization of F127@PDA@Fl. UV-Vis spectra of F127@PDA@Fl_40, F127@PDA@Fl_60 and F127@PDA_100@Fl (A). Fluorescence spectra of F127@PDA@Fl_40, F127@PDA@Fl_60 and F127@PDA_100@Fl in water (λ_{ex}= 492 nm) (B). UV-Vis spectra of F127@PDA_40 and F127@PDA@Fl_40 in water (C). Calibration curve for F127-TEG-NH_2 measured in water (D).

Table S3. DLS and zeta potential measurements of **F127@PDA** samples before and after functionalization with Fluoresein-TEG-NH_2. Errors are standard deviations of the triplicate data.

Sample	Hydrodynamic diameter [nm]	PDI	ζ [mV]
**F127@PDA_40**	59.2±0.9	0.027	-18.3±2.1
**F127@PDA@Fl_40**	71.6±1.7	0.116	-8.8±2.1
**F127@PDA_60**	75.2±1.1	0.040	-13.3±0.7
**F127@PDA@Fl_60**	79.4±3.2	0.132	-11.6±0.5
**F127@PDA_100**	117.4±2.2	0.016	-14.6-±1.6
**F127@PDA@Fl_100**	123.6±1.6	0.128	-9.4±1.3

2. *In vitro* evaluation of F127@PDA NPs

A

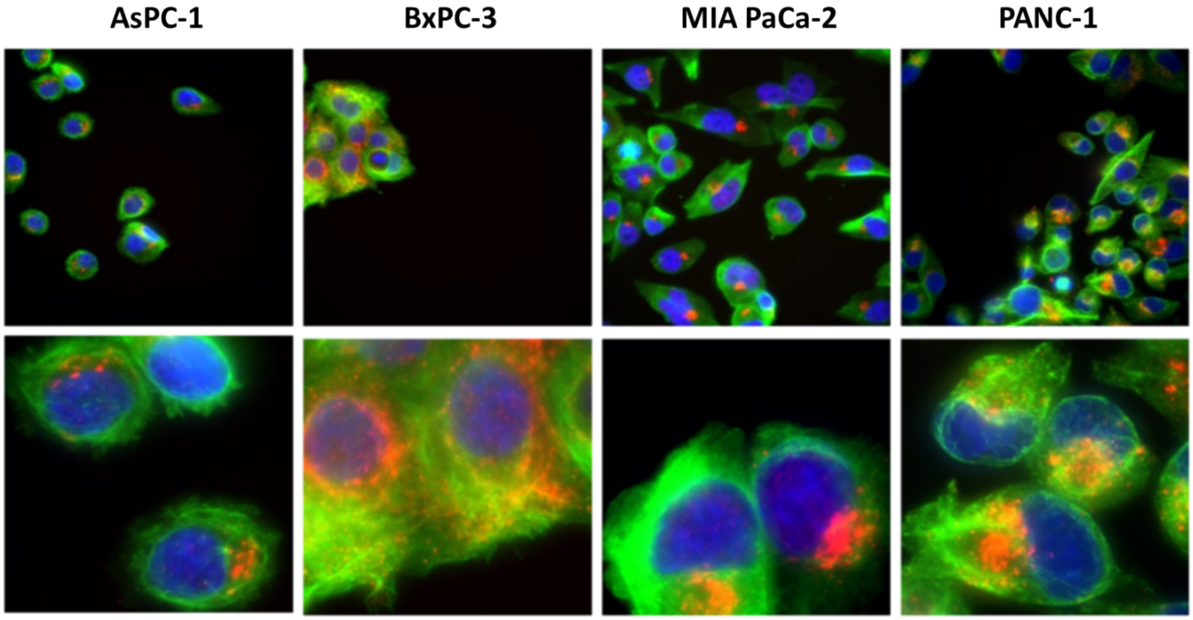

B

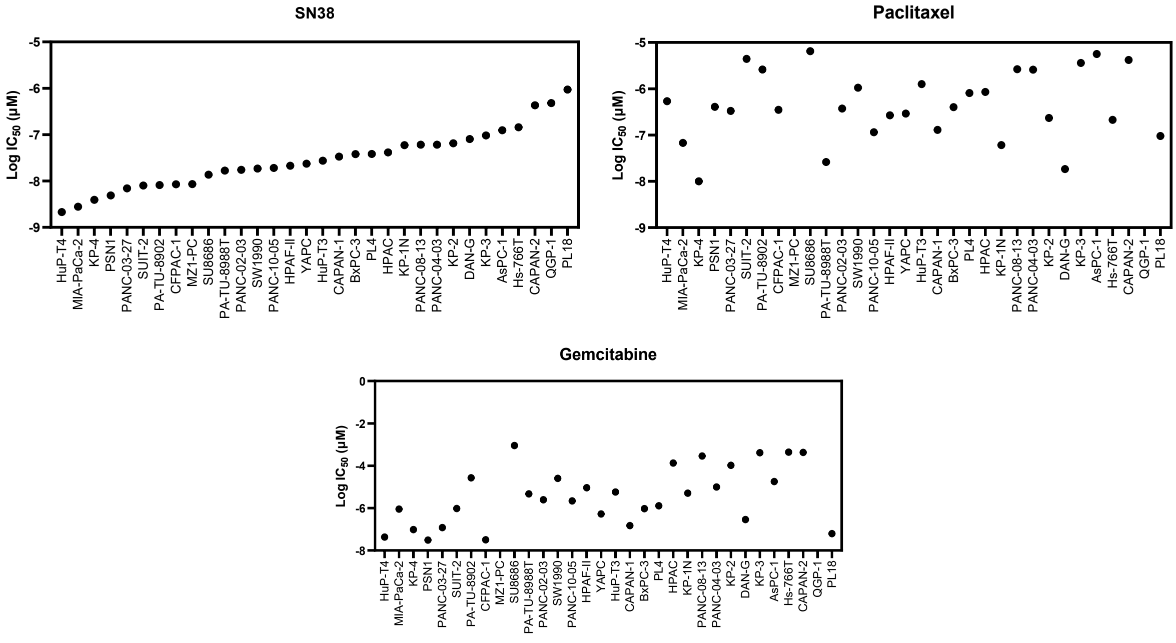

Figure S11. Endocytic profiling within different PDAC cells: nuclei (blue stain), tubulin (green stain), Golgi (red stain) (A). Summary of IC_{50} values obtained from Sanger drug screening data (https://www.cancerrxgene.org/)[3] for SN-38, Paclitaxel and Gemcitabine (B).

2.1. Cytotoxicity studies

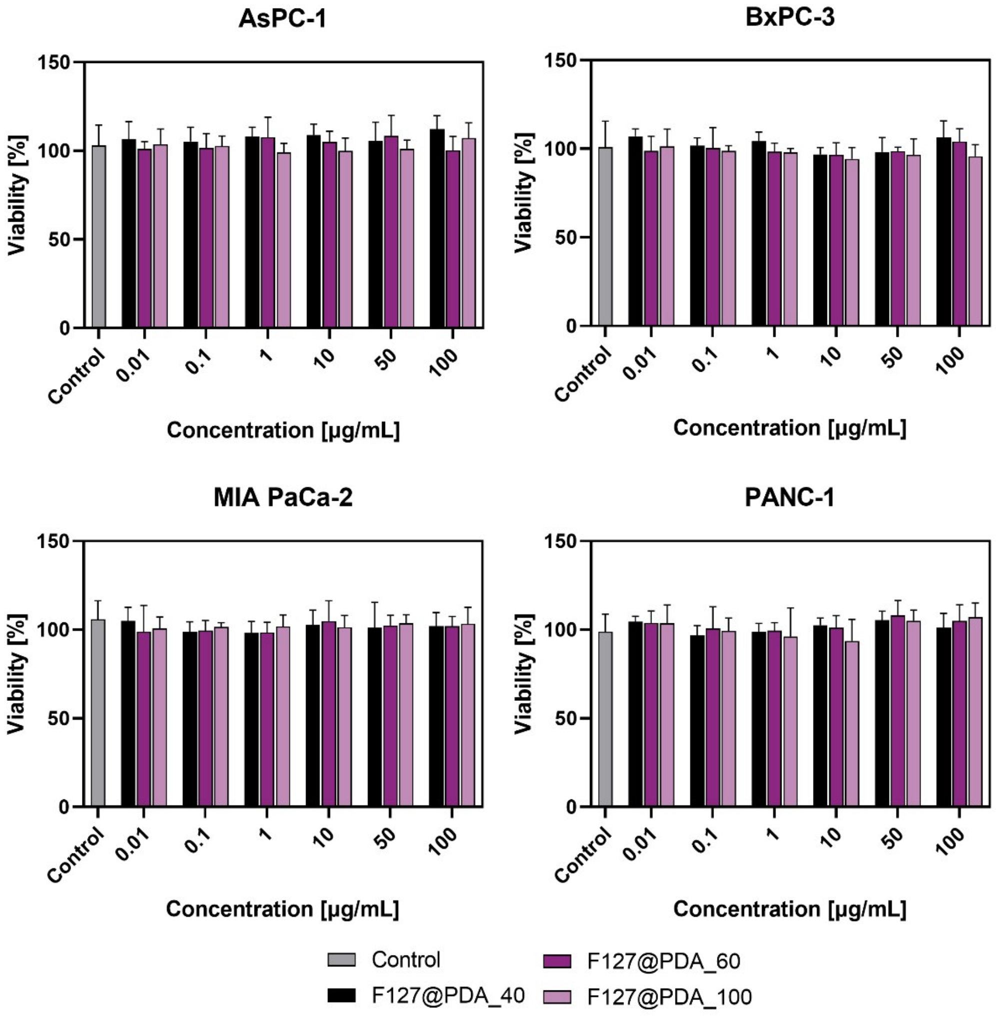

Figure S12. In vitro cytotoxicity effect of **F127@PDA_40**, **F127@PDA_60** and **F127@PDA_100** on AsPC-1, BxPC-3, MIA PaCa-2 and PANC-1 after 72 h incubation determined by MTS assay. Data are expressed as the mean ± SD.

2.2. Cell internalization studies

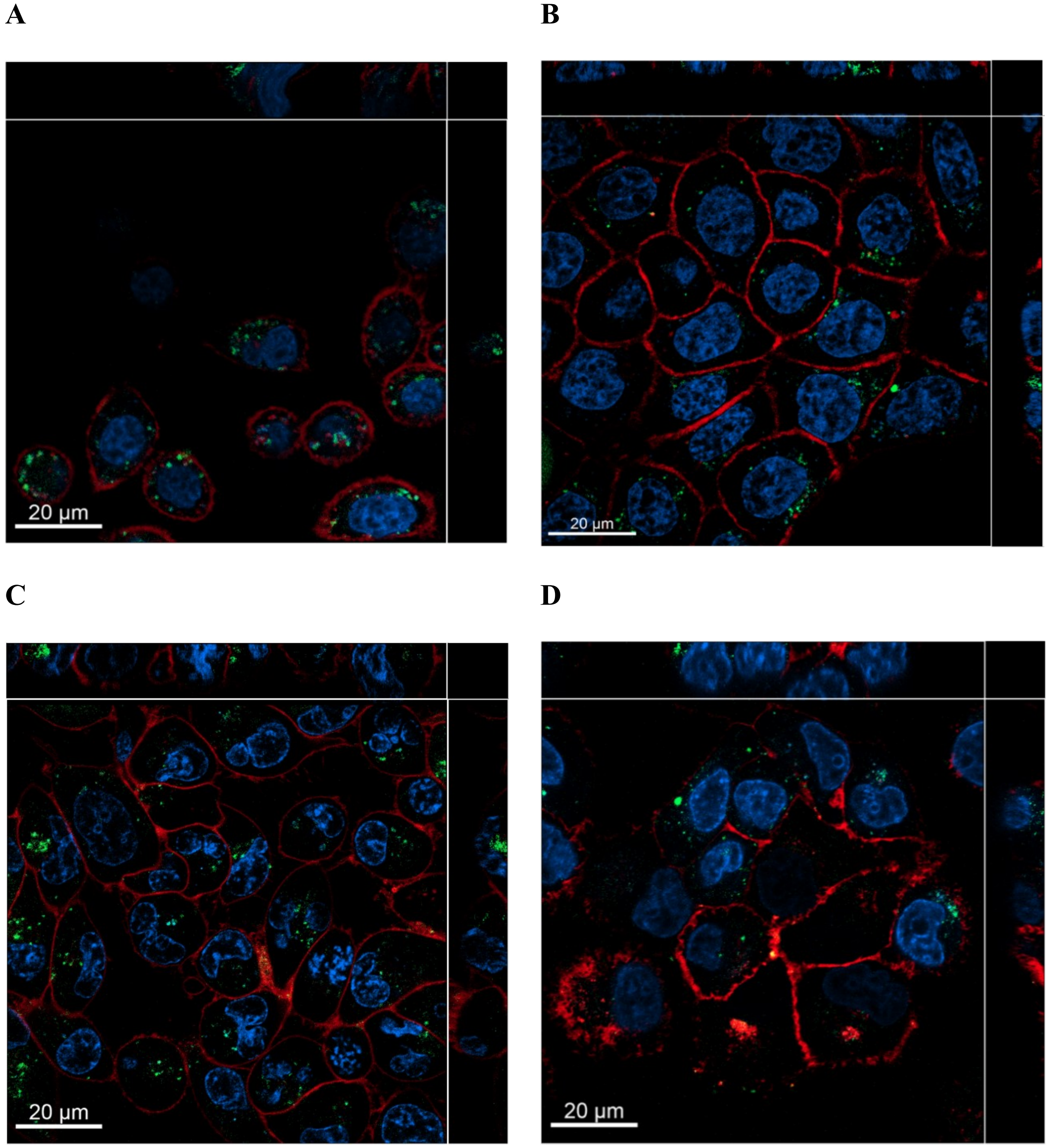

Figure S13. Orthogonal z-stack images of AsPC-1 (A), BxPC-3 (B), MIA PaCa-2 (C) and PANC-1 (D) after 24 h incubation with 50 µg/mL **F127@PDA@Fl_40** NPs acquired with confocal microscopy. Frontal view represents X–Y direction, top panel X–Z direction and right panel Y–Z direction. Cells were incubated with Cell Mask (deep red) and Hoechst 33342 (blue) to stain cell membrane and nuclei, respectively. Green dots represent the NPs.

A

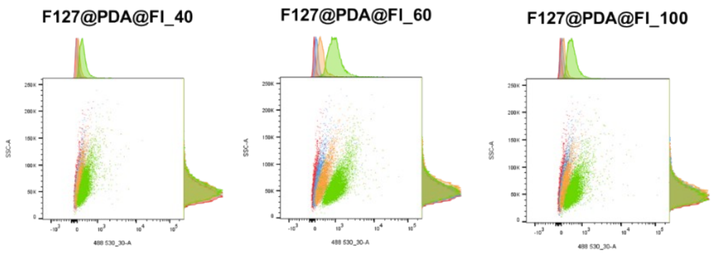

B

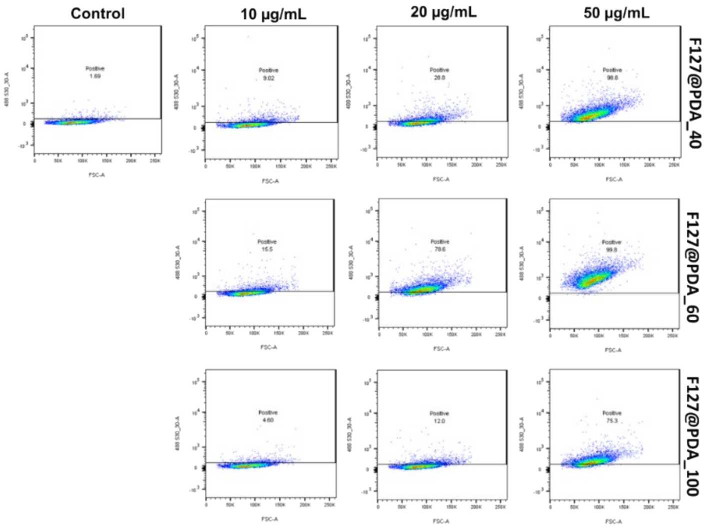

C

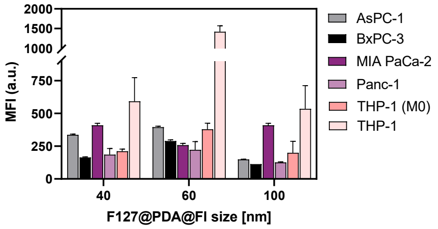

Figure S14. **Quantification of F127@PDA uptake using flow cytometry**. Effect of NP treatment on the side scatter of BxPC-3 cells (A). Example of FSC vs FITC graph for BxPC-3 after treatment with F127@PDA of different sizes with different concentrations (10, 20 and 50 μg/mL) used to calculate percentage of cells containing **F127@PDA@Fl** NPs in different cell types (B). Histogram showing normalized mean fluorescence values of AsPC-1, BxPC-3, MIAPaCa-2, PANC-1 THP-1 (M0) and THP-1 cells treated with 50 μg/mL **F127@PDA@Fl_40**, **F127@PDA@Fl_60** and **F127@PDA@Fl_100** for 24 h. MFI are represented as mean values and standard deviations of triplicate experiments (C).

2.3. Immunomodulation studies

A

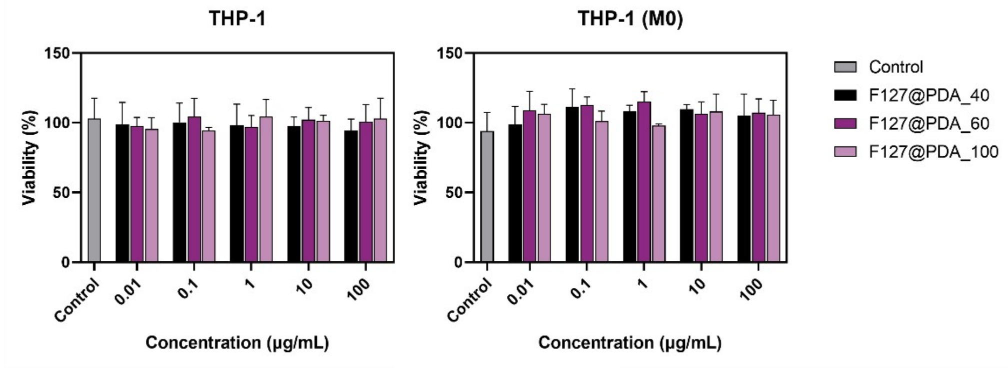

B

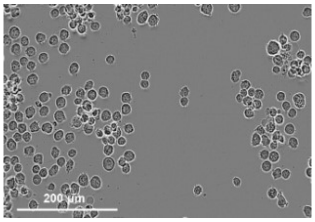

C

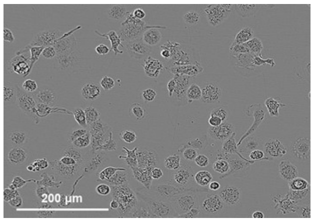

Figure S15. In vitro cytotoxicity effect of **F127@PDA_40**, **F127@PDA_60** and **F127@PDA_100** on THP-1 and THP-1 (M0) cells after 72 h incubation determined by ~~live~~ MTS assay (A). Data are expressed as the mean ± SD. Microscopic images of monocyte-like THP-1 (B) and PMA differentiated macrophages THP1 (M0) (C).

2.4. Drug loading and release studies

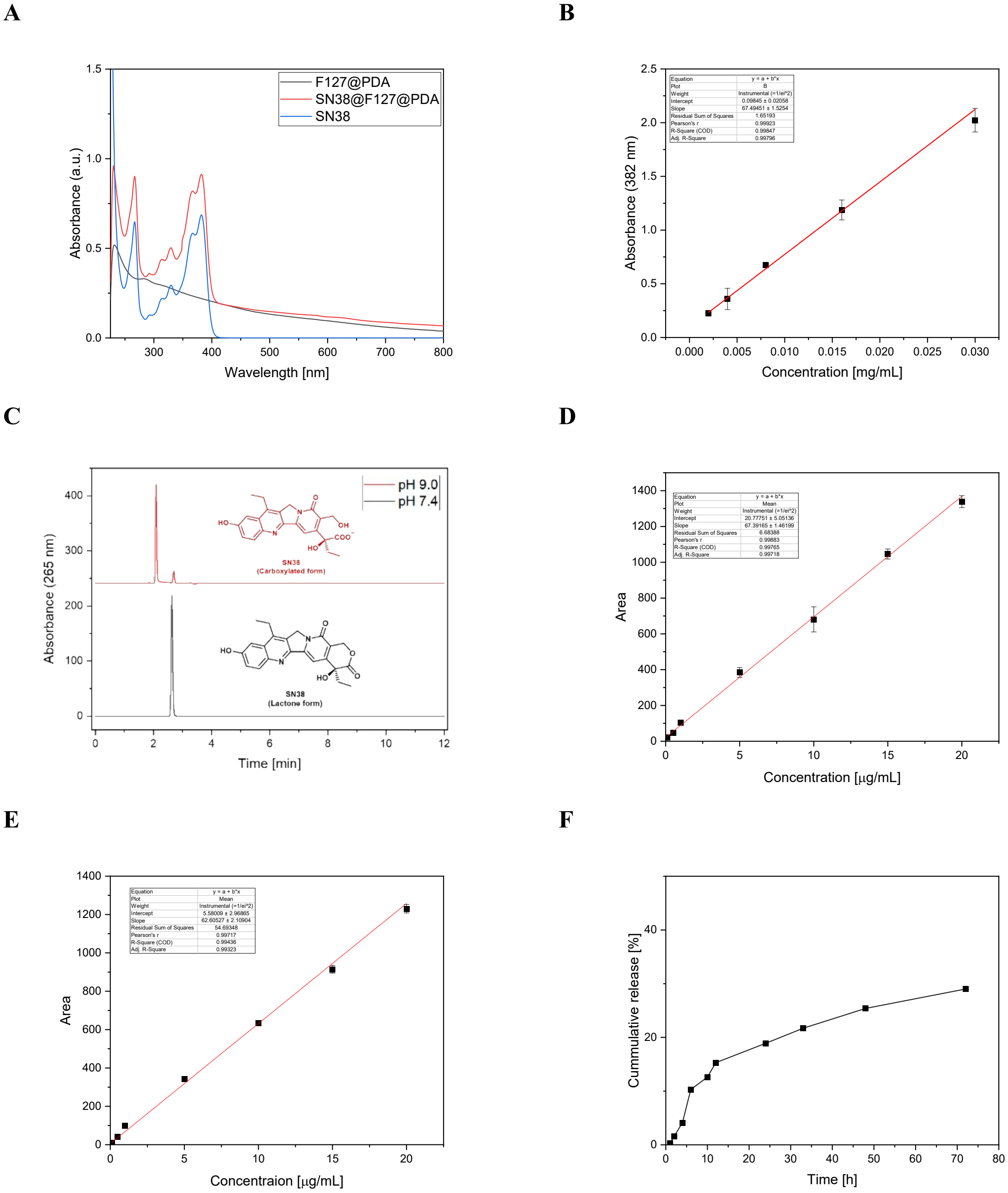

Figure S16. Drug loading of SN38. UV-Vis spectra of **F127@PDA**, **SN38** and **SN38@F127@PDA** (A) and calibration curve of SN-38 (B) measured in methanol using a 1 mL quartz cuvette. Example of HPLC spectra showing both lactone and carboxylate form of SN38. (C) Calibration curve of SN38 obtained for lactone (D) and carboxylate form (E) using HPLC. Cumulative release of SN38 in PBS (1X, pH = 7.4) during 72 h incubation at 37 ºC (F).

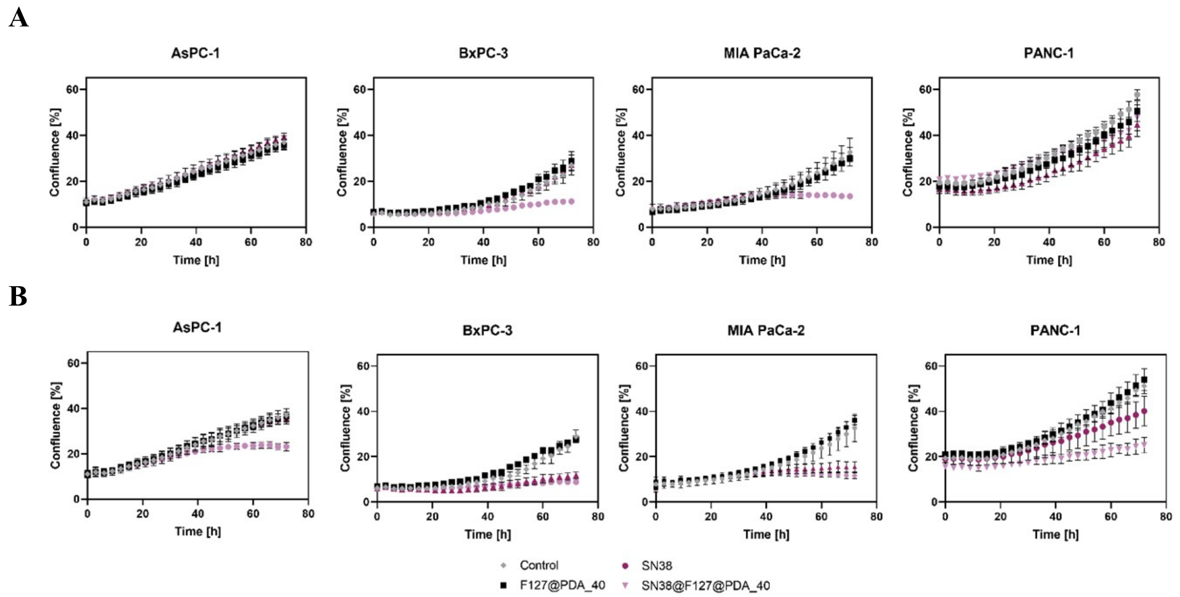

Figure S17. Growth curves of AsPC-1, BxPC-3, MIA PaCa-2 and PANC-1 obtained by live cell imaging during 72 h treatment with 1 nM (A) and 10 nM (B) SN38, **SN38@F127@PDA** and **F127@PDA**.

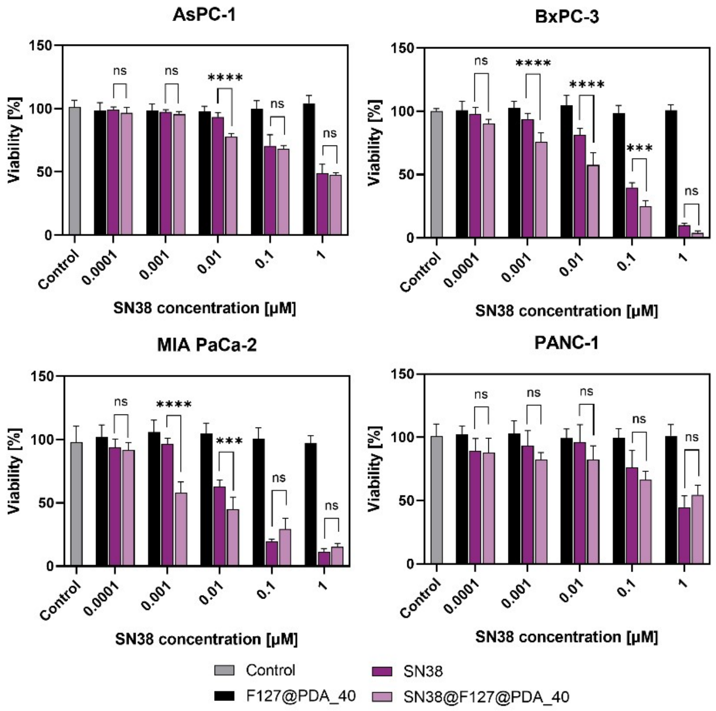

Figure S18. Cytotoxicity of SN38 and **SN38@F127@PDA** and **F127@PDA NPs** determined by MTS assay imaging after 72 h treatment of AsPC-1, BxPC-3, MIA PaCa-2 and PANC-1.

Table S4. IC_{50} values of **SN38@F127@PDA** and SN38 after treatment of AsPC-1, BxPC-3, MIA PaCa-2 and PANC-1 for 72 hours obtained from the dose-response by live cell imaging and MTS assay.

	Live cell imaging		MTS	
**Cell line**	IC_{50} (SN38) [nM]	IC_{50} (**SN38@F127@PDA**) [nM]	IC_{50} (SN38) [nM]	IC_{50} (**SN38@F127@PDA**) [nM]
**AsPC-1**	52.92	2.87	109.8	48.44
**BxPc-3**	8.91	< 0.1	55.96	11.37
**MIA PaCa-2**	12.11	<0.1	13.10	2.71
**PANC-1**	56.13	7.82	112.8	75.7

3. References

(1) Li, M.; Jiang, W.; Chen, Z.; Suryaprakash, S.; Lv, S.; Tang, Z.; Chen, X.; Leong, K. W. A Versatile Platform for Surface Modification of Microfluidic Droplets. *Lab Chip* **2017**, *17* (4), 635–639. https://doi.org/10.1039/c7lc00079k.

(2) Zeng, Z.; Mizukami, S.; Kikuchi, K. Simple and Real-Time Colorimetric Assay for Glycosidases Activity Using Functionalized Gold Nanoparticles and Its Application for Inhibitor Screening. *Anal. Chem.* **2012**, *84* (21), 9089–9095. https://doi.org/10.1021/AC301677V.

(3) Yang, W.; Soares, J.; Greninger, P.; Edelman, E. J.; Lightfoot, H.; Forbes, S.; Bindal, N.; Beare, D.; Smith, J. A.; Thompson, I. R.; Ramaswamy, S.; Futreal, P. A.; Haber, D. A.; Stratton, M. R.; Benes, C.; McDermott, U.; Garnett, M. J. Genomics of Drug Sensitivity in Cancer (GDSC): A Resource for Therapeutic Biomarker Discovery in Cancer Cells. *Nucleic Acids Res.* **2013**, *41* (D1), D955–D961. https://doi.org/10.1093/NAR/GKS1111.
